# The Epithelial-to-Mesenchymal Transition as a Possible Therapeutic Target in Fibrotic Disorders

**DOI:** 10.3389/fcell.2020.607483

**Published:** 2020-12-21

**Authors:** Jacopo Di Gregorio, Iole Robuffo, Sonia Spalletta, Giulia Giambuzzi, Vincenzo De Iuliis, Elena Toniato, Stefano Martinotti, Pio Conti, Vincenzo Flati

**Affiliations:** ^1^Burnett School of Biomedical Sciences, College of Medicine, University of Central Florida, Orlando, FL, United States; ^2^Institute of Molecular Genetics, National Research Council, Section of Chieti, Chieti, Italy; ^3^Department of Clinical Pathology, E. Profili Hospital, Fabriano, Ancona, Italy; ^4^Department of Medical and Oral Sciences and Biotechnologies, University “G. d’Annunzio”, Chieti, Italy; ^5^Postgraduate Medical School, University of Chieti-Pescara, Chieti, Italy; ^6^Department of Biotechnological and Applied Clinical Sciences, University of L’Aquila, L’Aquila, Italy

**Keywords:** EMT, fibrosis, autophagy, inflammation, TGF-β, SMADs, Wnt

## Abstract

Fibrosis is a chronic and progressive disorder characterized by excessive deposition of extracellular matrix, which leads to scarring and loss of function of the affected organ or tissue. Indeed, the fibrotic process affects a variety of organs and tissues, with specific molecular background. However, two common hallmarks are shared: the crucial role of the transforming growth factor-beta (TGF-β) and the involvement of the inflammation process, that is essential for initiating the fibrotic degeneration. TGF-β in particular but also other cytokines regulate the most common molecular mechanism at the basis of fibrosis, the Epithelial-to-Mesenchymal Transition (EMT). EMT has been extensively studied, but not yet fully explored as a possible therapeutic target for fibrosis. A deeper understanding of the crosstalk between fibrosis and EMT may represent an opportunity for the development of a broadly effective anti-fibrotic therapy. Here we report the evidences of the relationship between EMT and multi-organ fibrosis, and the possible therapeutic approaches that may be developed by exploiting this relationship.

## Introduction

**FIGURE 1:**
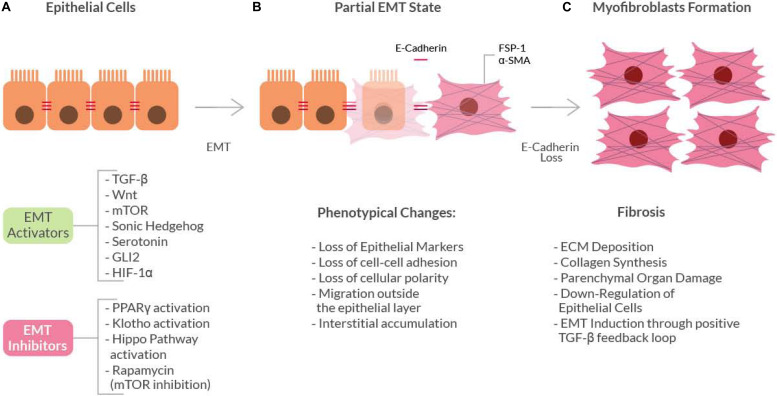
Schematic diagram showing the basic mechanism of the Epithelial-to-Mesenchymal Transition (EMT). Epithelial cells **(A)** can undergo EMT when stimulated by EMT-activators. These activators induce initial phenotypical changes (gray box) that leads to a ‘partial EMT’ state **(B)**, in which both epithelial and mesenchymal markers are expressed. The continuous pro-EMT stimuli can ultimately cause the loss of E-cadherin thus inducing the epithelial cells to acquire a full myofibroblast phenotype **(C)** and ultimately the fibrotic phenotype.

Fibrosis is characterized by an uncontrolled and excessive deposition of extracellular matrix (ECM) components. The increased ECM deposition then evolves to scar tissue formation and to a loss-of-function of the affected organ (skin, kidneys, lungs, cardiovascular system, liver, pancreas, intestine) (Leask and Abraham, 2004). The cell types mainly responsible for the ECM deposition, that leads to fibrosis are the myofibroblasts (Gabbiani, 2003). During normal wound healing, myofibroblasts undergo apoptosis when epithelialization has completed. On the contrary, in the pathological scenario, myofibroblasts persist and continue to synthesize collagen, leading to the fibrotic degeneration. Sources of fibroblasts for the fibrotic process also include: migration from nearby areas, proliferation and differentiation of stellate cells and recruitment from the bone marrow. However, there is a mechanism of fibrosis that involves differentiation and transformation of non-mesenchymal origin cells, a process called Epithelial to Mesenchymal Transition (EMT). This process involves epithelial cells that under certain stimuli undergo multiple biochemical changes and acquire a fibroblast phenotype (Kalluri and Weinberg, 2009), by losing their typical properties such as apical-basal polarity and cell-cell adhesion (Gabbiani, 2003; Inoue et al., 2015) (Figure 1). Depending on the organ where the fibrotic disorder occurs, EMT acts differently as a source of myofibroblasts.

Epithelial-to-Mesenchymal Transition may be classified into three ‘types.’ This classification takes into account the context in which the epithelial-to-mesenchymal transition occurs (Kalluri and Weinberg, 2009). Type 1 occurs during normal organogenesis, while type 2 occurs in the context of the healing process after injury: if the injury is mild and acute, the healing process is regarded as reparative fibrosis. On the contrary, in ongoing chronic inflammation conditions, the abnormal formation of myofibroblasts causes progressive fibrosis, thereafter leading to parenchymal organs damage by excessive ECM deposition. Type 3, on the other hand, is related to malignancy, where neoplastic cells can invade the surrounding tissues and migrate at metastasis sites and this type of EMT occurs in carcinomas derived from epithelial cells, in which neoplastic epithelial cells are transformed into cells with mesenchymal phenotype.

In wound repair myofibroblasts are located within the granulation tissue and exhibit a cytoplasmic microfilamentous system, which contains actin and myosin, as well as associated proteins of the smooth muscle, in particular alpha-smooth muscle actin (α-SMA), a differentiation marker of these cells. They are derived from fibroblast differentiation induced by growth factors, such as Transforming Growth Factor beta (TGF-β) or Platelet-Derived Growth Factor (PDGF), secreted by the epithelial cells in the wounded or inflamed area (Gabbiani, 2003). EMT, in fact, is caused and regulated by a complex signaling network: in a chronic inflammation scenario TGF-β1, oxidative stress and hypoxia activate a signaling cascade that leads to activation and stabilization of the transcription factor SNAIL, a well-known EMT activator (Xu et al., 2009).

EMT has been found to be associated with fibrosis in kidney, liver, lung, intestine and other organs (Rieder et al., 2011; Lamouille et al., 2013; Inoue et al., 2015). The first proof of principle came from the study of mouse models of fibrosis, where some of the isolated myofibroblasts exhibited the expression of epithelial cell-specific markers, such as E-cadherin, but they also expressed typical myofibroblasts markers, like the Fibroblast-Specific Protein 1 (FSP1) and α-SMA (Gabbiani, 2003). These findings indicated that, in EMT, epithelial cells slowly gain myofibroblasts markers as they lose their epithelial elements (cell expressing both types of markers are considered to be in a ‘partial EMT’ state). These cells then leave the epithelial layer, due to E-cadherin loss, and accumulate in the interstitium where they acquire a full myofibroblast phenotype.

An EMT-related mechanism of cell transformation that is also involved with fibrosis is called EndoMT (Endothelial to Mesenchymal Transition) (Piera-Velazquez et al., 2011; Rieder et al., 2011). Similarly, to EMT, the EndoMT is triggered by TGF-β and other proinflammatory cytokines like Tumor Necrosis Factor alpha (TNF-α) and Interleukin 1 (IL-1). *In vitro* and *in vivo* studies have shown that the major morphological changes of the endothelial cells are indeed triggered by the action of these three cytokines. It has also been observed that in presence of an inflammatory stress, EndoMT may contribute to the increase of fibrogenesis.

Here, we review the basis of the pathophysiological mechanisms of EMT, their regulation, and the roles played by EMT in fibrotic disorders that may allow the development of new therapeutic strategies.

## EMT and the Main Mechanisms of Fibrosis

### TGF-β and the Canonical Signaling

**FIGURE 2:**
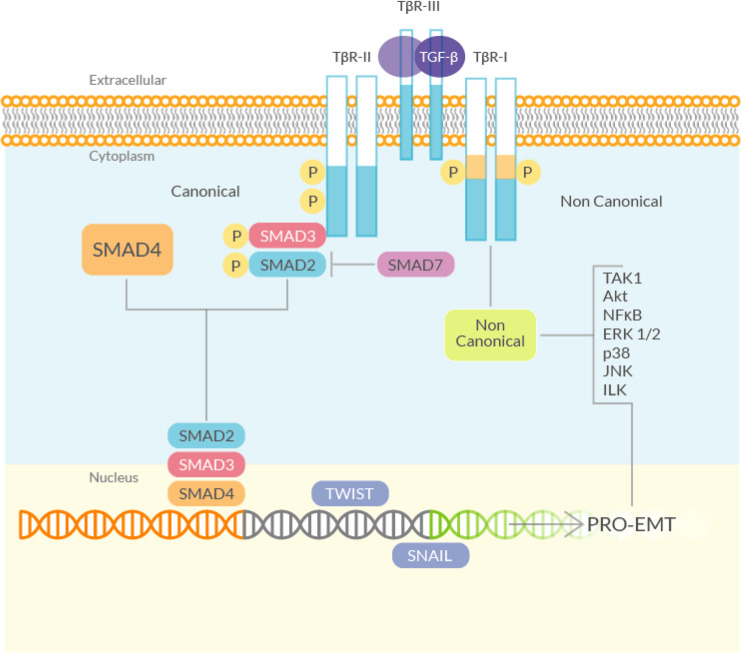
Schematic diagram of the TGF-β signaling. (Left) The canonical (Smad) signaling involves the TGF-β-Receptor, which phosphorylates R-Smads (Smad2 and 3). Smad2/3 bind to Smad4, translocate to the nucleus and activate, among others, genes that directly regulate EMT. Smad7, negatively controls the TGF-β signaling by inhibiting R-Smads phosphorylation, by competing directly with Smad2 and 3 for the TGF-β-Receptor binding. (Right) The non-canonical (non-Smad) TGF-β signaling pathway starts from the TGF-β-Receptor and mediates the induction of EMT through the activation of several kinases.

Transforming growth factor-beta is the main growth factor involved in fibrosis (Leask and Abraham, 2004) (Figure 2). TGF-β is rapidly induced after an injury and attracts macrophages and fibroblasts that release more of this growth factor; therefore, TGF-β is able to sustain itself through an autocrine loop. Like all the members of the TGF-β superfamily (nearly 30 proteins), it is a dimeric protein, synthesized as a latent precursor bound to other proteins called Latent TGF-β Binding Proteins (LTBP) and latency-associated peptide (LAP). TGF-β becomes activated when the bound proteins are detached (Robertson and Rifkin, 2016) and binds to a heterodimeric tyrosine kinase receptor (composed of the type I and type II TGF-β-Receptor subunits) to transduce the signal. The ‘canonical’ TGF-β signaling pathway involves the activation of the Smads, a family of transcriptional activator proteins. The TGF-β-Receptor directly phosphorylates the R-Smads (Receptor-activated Smads), Smad2 and 3, that bind to the common Smad mediator, Smad4, and translocate to the nucleus (Leask and Abraham, 2004). Smad3 is required for TGF-β-induced gene expression, while Smad2 is necessary for the normal development and seems to be also involved in the regulation of the, TGF-β-induced, expression of the pro-fibrotic matrix metalloproteinase 2 (MMP2) (Denis et al., 2016). Smad3 plays also a key role in the TGF-β mediated induction of other important signaling mediators such as c-fos and Smad7 (Piek et al., 2001). The latter is part of another group of Smad proteins, called the ‘inhibitory Smads’, that includes Smad6. They control the TGF-β signaling by inhibiting R-Smads phosphorylation (Yan et al., 2009). This inhibitory activity is mediated especially by Smad7 by competing directly with Smad2 and 3 for the TGF-β-receptor binding. TGF-β itself can induce Smad7, suggesting an auto-regulating mechanism, at least in normal conditions (Brodin et al., 2000).

The role of TGF-β signaling in EMT is well established: adding TGF-β to epithelial cell cultures results in a decreased expression of epithelial markers and an enhanced expression of mesenchymal markers: the typical signature of EMT. Induction of both Smad2 and Smad3 is associated with increased EMT, and KO of Smad3 prevents EMT induction in response to TGF-β stimulation, demonstrating the critical role played by the Smads in the TGF-β-induced EMT (Xu et al., 2009).

### TGF-β and the Non-canonical Signaling

In addition to the ‘canonical’ signaling pathway, TGF-β can also signal through “non canonical”, non-Smad, pathways (Yamada et al., 2013), that may also promote EMT. Phosphorylation of tyrosine residues in the activated TGF-β-Receptor results in the recruitment of several adaptor proteins, implicated with the activation of pathways that can mediate TGF-β-induced EMT, that are able to enhance the canonical TGF-β signaling (Medina et al., 2011). Indeed, various studies have shown that the ERK/MAPK pathway is required for the TGF-β induced EMT (Grände et al., 2002; Uttamsingh et al., 2008; Park et al., 2017). The activated TGF-β-Receptor can also act directly on myofibroblasts, thus promoting fibrosis. In an experimental model of intestinal fibrosis, it has been demonstrated that TGF-β type I receptor inhibition reduces Smad2 and 3 phosphorylation, collagen synthesis and TIMP-1 activity (which has been shown to exert a pro-fibrotic role in the gut). In this model the receptor itself is upregulated, suggesting that it plays an active role in the fibrotic process (Medina et al., 2011).

Another non-Smad signaling pathway involves the TGF-β activated kinase TAK1 (Transforming growth factor beta-Activated Kinase (1). TAK1 is a serine/threonine kinase that is rapidly activated by TGF-β but it can also be activated by environmental stress. This kinase can transduce signals to several downstream molecules (Choi M.E. et al., 2012). In particular, TAK1 has been shown to be associated with fibrosis in cultured fibroblasts where it was able to mediate the TGF-β induced expression of collagen I and IV (Strippoli et al., 2012). But, more interestingly, TAK1 phosphorylation was also found to be related to increased EMT, making TAK1 a candidate target for possible anti-fibrosis therapies (Gardner et al., 2012).

The link between EMT and TGF-β activity has also been demonstrated in mice models overexpressing the Bone Morphogenetic Protein 7 (BMP7), an antagonist of the TGF-β1 signaling pathway. BMP7 reversed the loss of E-cadherin induced by TGF-β via the canonical Smad pathway, and exogenous systemic administration of BMP7 managed to revert EMT inducing the repair of the damaged epithelia. These observations were later confirmed in human studies (Xu et al., 2009).

TGF-β is also known to cause the activation of the PI3K-AKT-mTOR signaling pathway, through which the EMT process can be enhanced (Lamouille et al., 2012). In fact, it has been shown that the inhibition of this pathway (by using rapamycin, a well-known mTOR inhibitor) attenuates EMT and the fibrotic processes (Bridle et al., 2009). Furthermore, an *in vitro* study has shown that TORC2 (mTOR complex 2) activity is also rapidly induced by TGF-β (via AKT phosphorylation), and activation of TORC2 is required for TGF-β-induced EMT (Cai et al., 2017). TORC2 regulates various aspects of the EMT, such as gene expression and cytoskeletal organization, and acts as a positive feedback regulator of the TGF-β signaling (Cheng and Hao, 2017). In addition, there is a number of *in vitro* studies that have underlined the link between the mTOR pathway and EMT in kidney, liver and lung fibrosis (Zhang D. et al., 2017; Zhu F. et al., 2017; Tan et al., 2017). These studies suggest that the mTOR pathway plays a pivotal role for the TGF-β-induced EMT in fibrosis.

The TGF-β signaling also crosstalk with another pathway implicated with EMT, the canonical Wnt/β-catenin pathway (Akhmetshina et al., 2012; Miao et al., 2013). The interplay between the two signals has been shown in wound healing and wound remodeling. In particular, the Wnt/β-catenin signaling regulates the effect of the TGF-β/Smad signals and, when activated, a prolonged β-catenin expression in hypertrophic scarring is observed (Liu et al., 2011). Furthermore, cells obtained from patients with hepatic and lung fibrosis, show a cross-talk between the two pathways during the fibroblast-to-myofibroblast transition (Chilosi et al., 2003; Guo et al., 2012). TGF-β was also found to upregulate β-catenin and Wnt signaling through the inhibition of the kinase Glycogen Synthase Kinase 3 beta (GSK3-β), thus enhancing the EMT process (Ye et al., 2013). Another study, on alveolar cells, has demonstrated that β-catenin is necessary for α-SMA (a hallmark of EMT) transcription, and its induction by TGF-β depends on CREB-binding protein (CBP, also known as cAMP response element-binding protein) activity (Henderson et al., 2010). In fact, an inhibitor (a small molecule called ICG-001) of the β-catenin/CBP interaction, reverted fibrosis and inhibited α-SMA and collagen induction (that are TGF-β dependent via Smad3 activation) (Villar et al., 2011; Zhou et al., 2012; Wei et al., 2012). The Wnt/β-catenin pathway plays another role in EMT induction. In fact, in normal conditions the inactive β-catenin forms a complex with E-cadherin located at the epithelial barrier (Tian et al., 2011). This complex is involved in maintaining the epithelial stability and in keeping silent the β-catenin signaling. In pathological conditions, such as fibrosis, the Wnt signaling is active, the complex is disrupted, the β-catenin is free to translocate into the nucleus, and the E-cadherin is degraded thus enhancing the loss of the epithelial phenotype. In fact, it has been shown that the EMT-mediated disruption of the E-cadherin/β-catenin complex leads to fibrosis. Furthermore, by inhibiting the complex disruption, the epithelial phenotype is restored, suggesting a pivotal role for the Wnt-mediated EMT in fibrosis (Di Gregorio et al., 2017).

In the fibrotic area, the EMT process is also induced by oxygen deprivation through TGF-β signaling. Hypoxia, in fact, is associated with various fibrotic diseases (Higgins et al., 2007, 2008; Haase, 2012) and TGF-β increased expression (Basu et al., 2011; Han et al., 2013). In fact, the key mediator of cellular response to hypoxia, the hypoxia-inducible factor (in both its isoforms HIF-1α and HIF-2), is induced and stabilized by TGF-β treatment and this results in increased fibrosis (Han et al., 2013). HIF-1α may also directly trigger EMT by modulating the expression of EMT regulators such as Snail, Slug (Romero et al., 2006; Grande et al., 2015) or Twist (Erler et al., 2006; Kida et al., 2007; Bechtel and Zeisberg, 2009). Another mechanism of Hypoxia-induced EMT involves the activation of LOXs (lysyl oxidases), that are HIF-induced genes capable to down-regulate E-cadherin expression and to enhance EMT (Erler et al., 2006; Trackman, 2016).

## EMT and Other Molecular Mediators of Fibrosis

**FIGURE 3:**
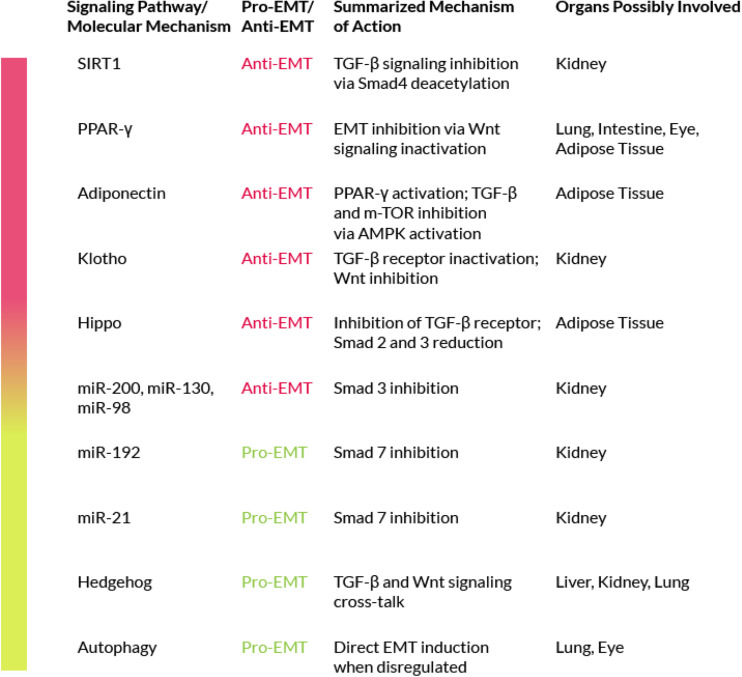
Signaling pathways/Molecular mechanisms that negatively or positively modulate EMT.

In addition to the TGF-β signaling and its crosstalk pathways, there are other molecular mechanisms that are involvedin both fibrosis and EMT (Figure 3).

### Peroxisome Proliferator Activated Receptor Family

The peroxisome proliferator activated receptors (PPARs) are key cellular mediators of fibrosis (Kulkarni et al., 2011). They are ligand-activated transcription factors that regulate a wide range of physiological activities and exist in three different subtypes: alpha, beta/delta and gamma. Activated PPARs and their co-receptors RXRs (Retinoid X Receptors) are able to bind a wide variety of ligands and play important roles in regulating processes related to fibrogenesis (Pirat et al., 2012). In particular, several studies have led to new insights into the PPAR-γ mechanism of action (Wei et al., 2010; Bertin et al., 2013) while other studies have investigated its anti-fibrotic properties (Speca et al., 2016). It has also been shown that PPAR-γ ligands, such as 15-Deoxy-Delta-12,14-prostaglandin J2 (15d-PGJ2), may counteract the pro-fibrogenic effects of TGF-β due to their ability to stop fibroblast-to-myofibroblast differentiation (Yang et al., 2010). In addition, PPAR-γ activation is able to revert EMT by acting through the inhibition of Wnt signaling (Di Gregorio et al., 2017). Furthermore, one of the transcriptional targets of PPAR-γ is adiponectin, an adipocyte-derived pleiotropic hormone with key protective roles in diabetes, atherosclerosis and other diseases. Sequence-specific recognition of the adiponectin gene promoter, the PPAR Response Element (PPRE), by activated PPAR-γ results in an enhanced adiponectin transcription. *In vitro* studies have underlined a possible anti-fibrotic activity for adiponectin. Indeed, the central modulator of adiponectin signaling is the AMP-activated protein kinase (AMPK), a key player also in cellular metabolism. Adiponectin, through AMPK activation, is capable of inhibiting the canonical TGF-β signaling. AMPK is also able to inhibit the mTOR signaling (which acts as an EMT activator), Fang et al. (2012). Taken together, these evidences support a role for adiponectin in EMT inhibition.

### The Hedgehog Protein Family

Members of the Hedgehog (Hh) family of proteins play a critical physiological role during embryo development; but the altered expression of several components of the Hh signaling pathway has been reported during experimental fibrogenesis (Cigna et al., 2012). Furthermore, *in vitro* studies have proven the cross-talk between Hh signaling and the canonical TGF-β pathway (Horn et al., 2012; Hu et al., 2015). TGF-β stimulation has been shown to reduce the mRNA levels of Hh-signaling inhibitors, such as Patched (Ptc), and to upregulate positive mediators of the same pathway (Stewart et al., 2003). These positive mediators are Smad3-dependent and have been observed in various cell types, including isolated colon cells from Crohn’s disease patients. TGF-β activates Gli2 (glioblastoma2), a mediator of Hh signaling, in a Smad3-dependent manner. Gli2 is also modulated by the Wnt/β-catenin pathway. Wnt and TGF-β cooperation leads to Gli2 upregulation and to the consequent Hh enhanced signaling, that further increases the fibrotic reaction via myofibroblasts activation (Dennler et al., 2009). In addition, in several studies the Hh pathway has been associated to the positive regulation of EMT in liver, kidney and lung fibrosis (Greenbaum, 2008; Omenetti et al., 2008, 2011; Choi et al., 2009; Syn et al., 2009; Bai et al., 2016; Lu et al., 2016). On these bases, the inhibition of the Hh pathway may represent a possible strategy to counteract EMT in fibrosis.

### The Hippo Pathway

The Hippo pathway is normally involved in organ size control but it also has other functions, including growth suppression, regulation of stress-induced apoptosis and of cell-fate (Imajo et al., 2012; Morgan et al., 2013; Carver and Goldsmith, 2013). Concerning the possible role of the Hippo pathway in EMT-related fibrosis, there is little information in the literature but on the base of the available data we can speculate that members of the Hippo pathway could be involved in the fibrotic disease for two main reasons. First, the Hippo pathway has been associated with EMT in cancer, in a study on MCF7 cells that showed that defects in the Hippo signaling result in Snail-induced EMT (Wang et al., 2016b). Snail is a family of transcriptional repressors that includes Snail1, 2 and 3 (the generic term Snail usually refers to Snail1). The Snail proteins are induced by the activation of the PI3K/AKT and Ras/MAPK pathways by several growth factors, including TGF-β, and act as molecular switches of the EMT program. The involvement of the Hippo signaling in EMT has been confirmed by a recent study, also in cancer cells, where it was shown that the inhibition of the Hippo pathway is required for EMT induction (Wang et al., 2016b). Second, the Hippo pathway is directly linked to processes that are proven EMT activators, such as the TGF-β signaling. However, this link has been found to depend on the cellular density. When it is low, TAZ and YAP (two of the downstream intracellular mediators of the Hippo signaling) are localized into the nucleus and promote Smad signaling via direct binding to the Smad2/3 complex. When instead the cellular density is high, the Hippo pathway is activated, and the two factors are localized in the cytoplasm, as their translocation into the nucleus is inhibited, and the binding to the Smads is blocked. YAP can also interact with Smad7, thus increasing its association with the type I TGF-β receptor that results in the inhibition of the TGF-β signaling (Grannas et al., 2015). Furthermore, defects of the Hippo pathway in malignant mesothelioma, synergize with TGF-β signaling to induce the common target CTGF (connective tissue growth factor) which results in matrix deposition, similarly, to what is observed in the fibrotic disease (Wang et al., 2016b). In addition, the Hippo pathway shows a crosstalk with Wnt signaling, being TAZ and YAP, the downstream effectors of Hippo, able to inhibit the Wnt/β-catenin pathway by interfering with β-catenin stabilization and activation (Deng et al., 2018). Phosphorylated YAP and TAZ interact also with the disheveled homolog (Dvl) and inhibit its activation through casein kinase 1 (CK1), thus inhibiting the pro-EMT activity of the Wnt signaling (Varelas et al., 2010). Furthermore, according to a recent study in kidney fibrosis, the use of a YAP inhibitor ameliorates renal fibrosis both *in vitro* and *in vivo* by decreasing the cellular levels of Smad2 and 3, thus the active YAP/TAZ complex cooperates with TGF-β to induce fibrosis in a Smad2/3-dependent manner (Szeto et al., 2016). These data suggest a precise role for the Hippo pathway in fibrosis-related EMT. Further studies on this pathway may provide new insights on the mechanisms of EMT in fibrosis and may allow the identification of targets for a possible anti-fibrotic therapy.

### MicroRNAs

MicroRNAs (miRNAs), are small non-coding RNAs around 20-25 nucleotides in length. miRNAs act as regulators of gene expression by inhibiting mRNA transcription via binding with its 3′ untranslated region (UTR). This binding is through an imperfect pairing thus a single miRNA can bind and can inactivate various mRNAs. MiRNAs have gathered the attention of many researchers in the last decade because they are virtually involved in all biological processes and may also play a role in various pathologies. Some of these miRNAs have a direct effect on EMT by either enhancing or inhibiting the process. Depending on the affected organ, there are miRNAs with a pro-EMT role and miRNAs with an anti-EMT role. Among the latter, miR-26a has a protective effect against EMT in the lung through a direct inhibition of TGF-β expression (Liang et al., 2016). Other miRNAs that inhibit EMT are mir-200, miR-130, or miR-98 and all of them are downregulated in lung fibrosis. The mir-30 and mir-200 families have been shown to play a protective role against kidney fibrosis. Their mechanisms of action include Smad3 inhibition, direct TGF-β inactivation and E-cadherin induction (Wang et al., 2016a; Yang et al., 2017b; Zhang N. et al., 2017; Zhang Q. et al., 2017). Among the pro-EMT miRNAs, mir-192 upregulates Smad3 and inhibits Smad7 in kidney fibrosis models (Chung et al., 2010). There are also other miRNAs that interfere with TGF-β, either by regulating its expression or through a feedback mechanism, being regulated by the factor itself, and thus may have an indirect effect on EMT. For instance, miRNA-21 becomes upregulated by TGF-β stimulation in various fibrosis models (Liu et al., 2010), resulting in Smad7 inactivation, ending with the activation of TGF-β signaling and directly enhancing EMT, as seen in lung fibrosis studies. Knockdown of miRNA-21 could therefore represent a possible anti-EMT strategy at least in lung fibrosis. However, there are also miRNAs with a more ‘general’ profile involved in the basic fibrotic mechanisms. The most important are those belonging to the mir-29 family, with its three members mir-29a, mir-29b and mir-29c. Down-regulation of the mir-29 family members is associated to enhanced fibrosis in various organs, whereas their induction (forced overexpression or drug-mediated) results in fibrosis inhibition. A recent study, in mice, has demonstrated the role played by mir-29b as an EMT inhibitor in lung fibrosis induced by silica, underlining the possible therapeutic use of mir-29 for silicosis (Liu et al., 2010; Sun et al., 2019). The precise mechanism of this anti-fibrotic activity is still under investigation, nevertheless, the mir-29 family seems to have a series of targets, from TIMP1 (that inhibits the MMPs and ECM degradation), to PDGFR (the receptor for PDGF, a key fibrotic mediator), that end in an anti-fibrotic effect. Thus, the mir-29 family, also known as ‘fibromiRNA,’ represents the main target for a possible anti-fibrotic therapy (O’Reilly, 2016). To deepen the knowledge about the interplay between miRNA and EMT, we refer you to a recent review (O’Reilly, 2016).

Taken together, these observations suggest the use of miRNAs as a therapeutic tool against the EMT-driven fibrosis. However, this approach might be complicated by the evidence, at least in the intestine, that microRNA mediated posttranscriptional regulation of gene expression also regulates autophagy (Wang S. et al., 2018).

### Autophagy

Macroautophagy (commonly, and hereafter in this text, referred to as autophagy) is an intracellular process that is essential for the clearance of unused long-lived proteins, damaged cytoplasmic proteins and organelles and through this mechanism the cytoplasmic components are degraded and recycled. In autophagy the target structures are engulfed in autophagosomes that then fuse with the lysosomes, forming the autophagolysosomes that degrade their content through the lysosomal hydrolases. Autophagy is regulated by a complex signaling network and its malfunctioning leads to several diseases including fibrosis. In particular autophagy alterations may result in a more severe fibrotic phenotype. Autophagy has also an EMT inducing effect on cancer cells (Liu et al., 2017) and it exerts a pro-fibrotic role, when activated, in lung fibrosis. Furthermore, several studies underline a connection between the activation of autophagy and intestinal fibrosis-associated EMT. Studies performed on colonic epithelial cells showed that an upregulated autophagy enhances the inflammatory state, that is required for the fibrotic reaction in the intestine, by enhancing the production of TNF-α (Xavier et al., 2008; Saito et al., 2012). Being TNF-α able to enhance the autophagy levels, at least in colon adenocarcinoma HT-29 cells, and to stimulate the loss of their adhesive capacity, an event occurring during EMT (Saito et al., 2012), it can be inferred that autophagy contributes to intestinal fibrosis induction. However, further studies are needed to better support this concept. A complication in targeting autophagy as an anti-fibrotic approach arises from the fact that the role of autophagy may be significantly different in the context of different fibrotic organs. In fact it may be insufficient and thus promotes the disease in idiopathic pulmonary fibrosis (IPF) (Araya et al., 2013) while it attenuates tubulointerstitial fibrosis in the kidney (Nam et al., 2019). Moreover, autophagy has been shown to exert mixed activities also in the liver fibrosis (Mao and Fan, 2015).

## Organ Fibrosis, EMT and Possible Anti-EMT Therapeutic Strategies

### Liver Fibrosis

Liver fibrosis generally occurs as a consequence of a deregulated wound healing, following liver damage (Bataller and Brenner, 2005). Hepatic fibrosis leads to hepatocellular dysfunction, hepatic insufficiency, portal hypertension and, at the end, to liver failure (Ebrahimkhani et al., 2011). In liver fibrosis, the hepatic stellate cells (HSC) are the main producers of the fibrotic tissue (Yanagida et al., 2011). In a state of chronic liver damage and inflammation, TGF-β triggers the differentiation of HSC into myofibroblasts, that in turn secrete more pro-fibrotic factors (Gieling et al., 2009; Mentink–Kane et al., 2011; Li et al., 2012). An additional source of myofibroblasts comes from the EMT process (Zeisberg M. et al., 2007; Zhao et al., 2016) whose involvement in liver fibrosis has been demonstrated in several studies (Inagaki et al., 2003; Seki et al., 2007; Feng et al., 2013; Li et al., 2017; Park et al., 2017) due to the activation of pro-EMT pathways, such as the TGF-β. In addition, the activation of this pathway is enhanced by the long noncoding RNA H19, found upregulated in murine fibrotic liver tissues. RNA H19 is a competitor of miR-148a, a member of the miR-148/152 family that is downregulated in liver fibrosis. Thus, targeting of RNA H19 may represent a novel therapeutic approach for the hepatic fibrosis (Zhu et al., 2019). In addition, the Wnt/β-catenin pathway, through its crosstalk with the TGF-β signaling, can upregulate myofibroblasts formation and their consequent fibrotic tissue deposition (Yanagida et al., 2011) and inhibition of the mTOR pathway by rapamycin attenuates hepatic fibrosis in rat models (Bridle et al., 2009). A further fibrogenic mechanism active in the liver is mediated by macrophages. These cells enhance the fibrotic reaction by interacting with myofibroblasts, via paracrine signaling, by increasing TGF-β1 secretion and by releasing pro-fibrotic MMPs (such as MMP9 and MMP2) (Zhu L. et al., 2017). In addition, macrophages secrete a wide variety of chemokines that further recruit myofibroblasts and inflammatory cells in the fibrotic tissue. In fact, in various models of liver fibrosis, chemokines such as Macrophage Inflammatory Proteins (MIP)1-alpha and beta and their receptors, CCR1 and CCR5, are upregulated. Their activity results in a more severe inflammatory phenotype, enhanced angiogenesis, and enhanced recruitment of macrophages and myofibroblasts further increasing fibrosis in the damaged liver (Kaviratne et al., 2004).

To date, there are no available data about the direct involvement of macrophages in EMT activation in the liver. However, according to several studies performed on tumors, macrophages are able to activate EMT, mainly via TGF-β induction (Zhang et al., 2015; Yang et al., 2016), thus it cannot be ruled out their possible contribution to EMT in the liver. Moreover, EMT is deeply implied in a particular type of fibrotic hepatic disorder, the cholangiopathies. Briefly, cholangiopathies are a group of liver disorders that target the epithelium of the bile ducts, the cholangiocytes (Lazaridis et al., 2004; Strazzabosco et al., 2005). In these diseases cholangiocytes acquire and exhibit mesenchymal markers, while losing epithelial properties, indicating that they are going through EMT, as observed in animal models and in patients (Rygiel et al., 2008; Harada et al., 2009; Sung et al., 2014). A study (Omenetti et al., 2008) has even proposed a specific mechanism that sees the Hh signaling as the pivotal EMT activator. These observations are strengthened by the augmented Hh signaling observed in several cholangiopathies (Jung et al., 2007; Syn et al., 2009; Omenetti et al., 2011; Grzelak et al., 2014) and by the enhanced levels of the Hh activator Gli2 observed in experimental models (Choi et al., 2009). In this particular pathological scenario, TGF-β mediated induction of Hh signaling (Rygiel et al., 2008) and the consequent EMT appear to be responsible of the fibrotic reaction. Furthermore, the overexpression of long noncoding RNA-maternally expressed gene 3 (LncRNA-MEG3) blocks HSC activation and inhibits the Hh-mediated EMT process. There is therefore a clear correlation between LncRNA-MEG3 and liver fibrosis that could be leveraged for therapeutic purposes (Yu et al., 2018). In hepatocyte EMT models, the therapeutic efficacy of baicalin, a bioactive agent extracted from Scutellariae Radix, and puerarin, an isoflavonoid extracted from the root of Pueraria Lobata, has been examined and it has been determined that they reverse EMT by reducing the mRNA levels of mesenchymal markers such as Snail, TGF-β1 and Smad3 and by increasing the expression of the epithelial marker E-cadherin (Wu et al., 2018). Baicalin therapy can be effective in alleviating liver fibrosis in rat models, with a reduction of hepatic hydroxyproline, alanine and aspartate aminotransferases, ALT and AST (the most commonly used liver injury markers), the reduction of TGF-β1, TNF-α and IL-6 levels and the abrogation of deposition and accumulation of collagen (Peng et al., 2009). Another natural compound that has been shown to be an EMT preventing agent is Ginsenoside Rg1 of Ginseng. It protects from liver fibrosis (Tsuda et al., 2013) since it reverses TGF-β-stimulated EMT, a fact demonstrated by the reduction of liver damage associated markers such as ALT and AST, of cellular collagen and reactive oxygen species (ROS) levels (Wei et al., 2018).

In liver fibrosis, the activation of HSCs is a crucial event because it leads to the development and progression of the disease. HSC activation can be stimulated by a variety of factors, such as the pro-fibrogenic TGF-β1. Thus, a combination therapy approach that targets TGF-β, its regulating pathways and the associated inflammatory state may represent an option for liver fibrosis treatment (Ebrahimkhani et al., 2011; Bai et al., 2013; Feng et al., 2013; Geng et al., 2020). In addition, other cytokines can stimulate the fibrotic process in the liver (Tsuda et al., 2013; Youssef et al., 2013). For instance, IL-1β exerts fibrogenic effects similar to TGF-β, by inducing EMT. *In vitro*, when IL-1β is inhibited by the human monoclonal antibody Canakinumab, the EMT markers (vimentin, α-SMA, fibronectin) result minimized. Thus, Canakinumab, by blocking IL-1β, has the potential to protect from hepatic fibrosis and likely from fibrosis of other organs (Masola et al., 2019).

**FIGURE 4:**
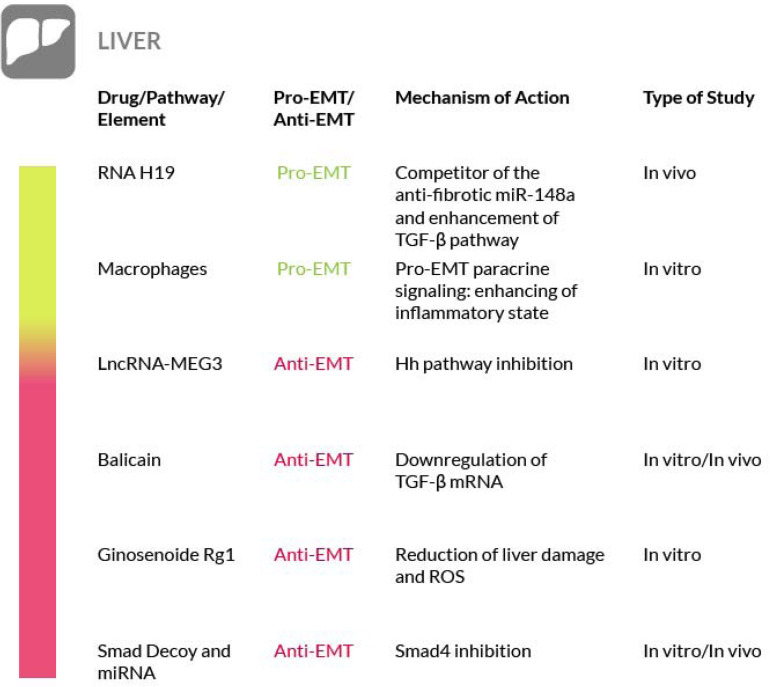
Most relevant molecular mechanisms and possible drugs for the treatment of EMT in liver Fibrosis.

A further strategy to prevent liver fibrosis contemplates the use of Smad decoy oligodeoxynucleotides, long noncoding RNAs and miRNAs (Gwon et al., 2018). Among them, several miRNAs have recently emerged as remarkable regulatory elements of fibrogenesis, although they can exert a pro-fibrotic or anti-fibrotic activity, depending on the affected organ. In particular, the overexpression of miR-146a, miR-122 and miR-30a resulted in the suppression of induced liver fibrosis accompanied by a decreased expression of EMT-associated proteins (Zhang Y. et al., 2018; Zou et al., 2019; Cheng et al., 2019). Overexpression of miR-146a preserved the hepatocytes against EMT by targeting Smad4 and thus by exerting an inhibitory activity towards the TGF-β1 signaling pathway. Similar results have also been obtained with other miRNAs such as miR-30a, that targets the Snail family of transcriptional repressor 1 (SNAI1) by causing its reduction (Zheng J. et al., 2018). miR-140-3p knockdown suppresses HSCs proliferation and fibrogenesis induced by TGF-β1 (Wu S.M. et al., 2019). Let-7a miRNA expression has been found decreased in mouse models of liver fibrosis and in the liver and blood samples from liver fibrosis patients. Let-7a suppresses the activation of HSCs and its overexpression induces apoptosis of these cells, by interfering with the TGF-β/SMAD signaling pathway (Zhang et al., 2019). Thus, the administration of miRNAs may represent an interesting therapeutic opportunity to combat liver fibrosis. Taken together, these observations suggest that the EMT process may be more involved in liver fibrosis than actually believed; however, further studies are needed to better support this concept and we have to underline the fact that in the literature the true contribution of EMT to liver fibrosis is still debated (Taura et al., 2016). Nevertheless, although in liver fibrosis, targeting of the pathways mediated by TGF-β, PDGF, Wnt/β-catenin and inflammatory factors are possible therapeutic strategies, their downregulation has been shown effective against the disease in various studies (Figure 4).

### Renal Fibrosis

Renal fibrosis can be defined as the ‘final stage’ of various inflammatory diseases of the kidneys such as tubule-interstitial fibrosis or glomerulosclerosis (LeBleu et al., 2013). These progressive diseases result in fibrotic tissue deposition in the kidneys, at the expense of the normal tissue, leading to a progressive loss of renal function (Lv et al., 2018). In chronic inflammation, a wide range of pro-fibrotic cytokines regulates inflammatory cells infiltration, mesangial cells activation, myofibroblast differentiation, cell apoptosis and ECM expansion. Thus, inflammation, although possibly involved in other organs’ fibrosis, is strongly related to renal fibrosis, in particular. Indeed, inflammatory aggregates of neutrophils, macrophages and lymphocytes have been observed in various models of renal fibrosis (Pan et al., 2015). After neutrophil infiltration, macrophages and lymphocytes recruit pro-fibrotic cytokines and chemokines that stimulate ECM deposition.

At the molecular level renal fibrosis has been extensively studied and TGF-β signaling has been identified as the main profibrotic mediator. In fact, TGF-β is released by the macrophages of the inflammatory infiltrates and it is found to be upregulated in all the chronic kidney diseases. A recent study (Pan et al., 2015) has shown that inhibition of macrophages activity drastically inhibits EMT, making the macrophages a potential therapeutic target in kidney fibrosis. Moreover, macrophages inhibit BMP7, that acts as a counterbalance of TGF-β signaling (Li et al., 2015; De Langhe et al., 2015), via Smad3 downregulation, thus acting as an EMT inhibitor (Wang et al., 2014; De Langhe et al., 2015). In kidney, TGF-β signaling also enhances other pro-EMT pathways. It is the case of the Wnt pathway, that has been shown to contribute to renal fibrosis, in in vivo and in vitro models (Satoh et al., 2012; Kawakami et al., 2013; LeBleu et al., 2013); of Nuclear Factor kappa-light-chain-enhancer of activated B cells (NF-κB) and Mitogen Activated Protein(MAP) kinases that further enhance the inflammatory state (Park et al., 2017); of mTOR, that has been associated with enhanced TGF-β expression (Cheng and Hao, 2017). Moreover, it has been reported (Omenetti et al., 2008; Bai et al., 2016) the involvement of the Hh pathway in TGF-β-induced EMT *in vitro* and *in vivo*, and the blockade of Hh reverted the myofibroblast phenotype, making this pathway a potential target for kidney fibrosis therapy.

In addition, several studies have underlined a critical role for hypoxia during the development of renal fibrosis, by mediating the EMT induction (Haase, 2012). Hypoxia-inducible factor (HIF-1α) and Lysyl oxidases (LOXs) are responsible for the increased EMT (Trackman, 2016) and ablation of HIF-1α and inhibition of LOXs could ameliorate renal fibrosis (Erler et al., 2006), making LOXs and HIF-1α possible targets for an anti-EMT and anti-fibrosis therapy in kidney. Considering that most of the molecular mechanism of fibrosis deregulation in the kidney is shared among the different fibrotic organs, it is conceivable that a valid anti-fibrotic strategy found for the kidney might also be translated to the treatment of other fibrotic organs (Lehmann et al., 2000; Park et al., 2017; Zhu F. et al., 2017). This hallmark of kidney fibrosis has also led to the idea that “drug repositioning” could be an interesting way to develop new anti-EMT therapies also effective in other organ fibrosis. For instance, an anti-fibrotic role for the well-known anti-cancer drug paclitaxel has been proposed, by reducing α-SMA and fibronectin via STAT3 inactivation. Furthermore, in TGF-β1-stimulated murine cells, paclitaxel treatment has been shown to inhibit Smad2/3, JNK and ERK1/2 activation, and improved kidney fibrosis (Jung et al., 2016). The concept of drug repositioning could be also applied to diabetes-related renal fibrosis. In fact, it is well established the link between EMT and diabetic kidney disease, where renal tubular EMT could be induced by high-glucose levels. Metformin, the oral drug commonly used for type 2 diabetes mellitus (T2DM) therapy, has been found to improve the renal function and the related EMT/tubulointerstitial fibrosis via AMPK activation and TGF-β1 downregulation. This latter effect results in the downregulation of the early growth response-1 (Egr-1) gene expression which plays a key role in tissue fibrosis. Thus, metformin may also exert an antifibrotic activity by reducing Egr-1 expression (Guan et al., 2018). Furthermore, several compounds from natural sources have been tested for their efficacy against renal fibrosis. Triptolide, the active component of Tripterygium wilfordii, is able to counteract the PI3K/AKT signaling induced by high-glucose levels and the overexpression of miR-188-5p. In diabetic kidney disease (DKD) animal and cell models, triptolide negatively regulates miR-188-5p, showing anti-EMT activity and protecting from renal interstitial fibrosis. The downregulation of this miRNA leads also to the amelioration of the high glucose-induced renal tubular EMT associated with DKD (Xue et al., 2018). Resveratrol, a natural stilbene, or phenol, found in red grapes and some berries, can ameliorate the renal function in a dose- and time of exposure- dependent manner (Liu S. et al., 2019). Other studies have demonstrated how baicalin, a type of flavonoid extracted from herbal roots of several species of the genus Scutellaria, including Scutellaria baicalensis, exerts a protective role against kidney fibrosis. Baicalin suppresses EMT via inhibition of TGF-β1 and the downstream signal cascade, including Smad2/3, as evidenced by changes of kidney morphology and of key EMT proteins expression (Zheng et al., 2017). In addition, the efficacy of Ribes Diacanthum Pall (RDP) extract has been investigated in a renal fibrosis model, by evaluating pro-inflammatory cytokines levels and activity of the TGF-β/Smad and MAPK pathways. The levels of p-Smad2/3 and p-ERK1/2 were found attenuated, as well as the levels of α-SMA, collagen I and fibronectin (Gu et al., 2019). Another natural product that exhibits anti-fibrotic effects is Ginsenoside-Rg1 of Panax ginseng. In rat models, it has been observed that it mitigates the EMT by suppressing TGF-β1 signaling and by upregulating Klotho and Smad7 expression (Li N. et al., 2018; Li S. et al., 2018). Finally, administration of asperulosidic acid, an iridoid glycoside isolated from Hedyotis diffusa, provided various beneficial effects in an unilateral ureteral obstruction model and it also protects from renal interstitial fibrosis, by reducing the level of inflammatory proteins and by downregulating TGF-β1 signaling (Xianyuan et al., 2019).

**FIGURE 5:**
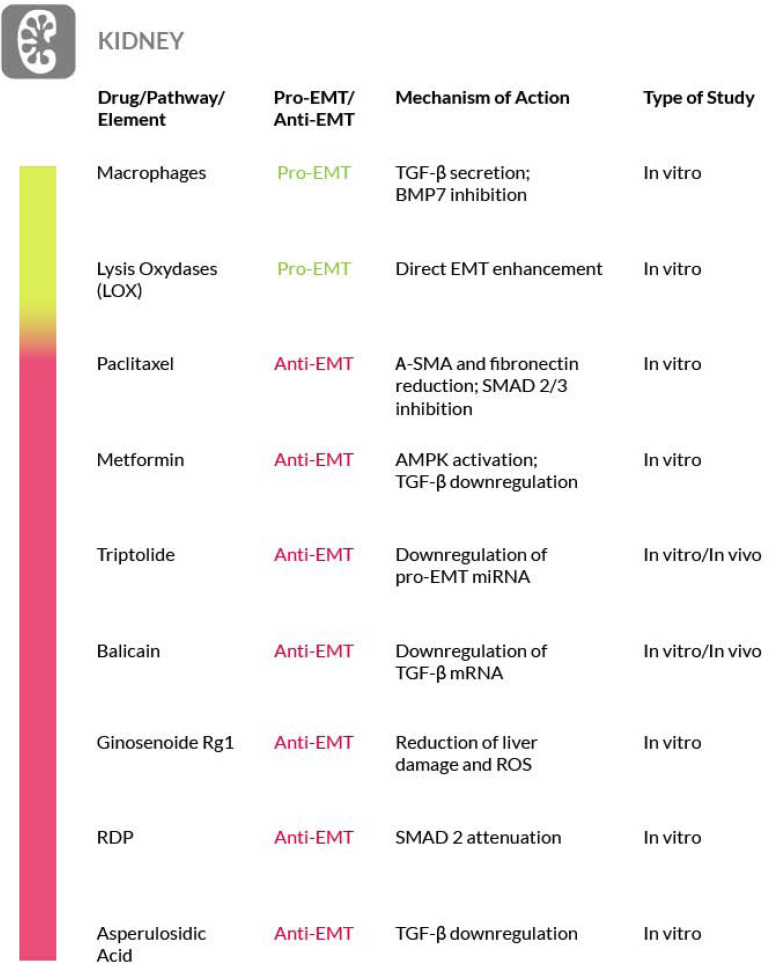
Most relevant molecular mechanisms and possible drugs for the treatment of EMT in renal fibrosis.

We must point out that the true involvement of EMT in kidney fibrosis need to be further substantiated. In fact, probably because of the variety of experimental models used, there is a certain amount of confusion in the literature. For a broad discussion on this matter we refer you to recent reviews (Cruz-Solbes and Youker, 2017; Sheng and Zhuang, 2020) (Figure 5).

### Pulmonary Fibrosis

The fibrotic reaction occurring in lungs can be of two types. The first, interstitial non-idiopathic fibrosis (PF), is a complication of distinct chronic pathologies or long-term exposure to damaging agents affecting the respiratory tract, Chen et al. (2001), Ding et al. (2013), and De Langhe et al. (2015). The second is the idiopathic pulmonary fibrosis (IPF), a progressive and fatal interstitial lung disease of unknown origin (Pandit et al., 2011; Pfaff et al., 2011; Sterclova and Vasakova, 2014; Raghu and Selman, 2015; Woodcock and Maher, 2015). In PF, inflammation has been identified as the main cause of the fibrotic reaction, leaving a secondary role to EMT (Chapman, 2011). In IPF, when inflammation is not involved, EMT may instead play a crucial role in the development of the pathology (Ohbayashi et al., 2014). Although the detailed mechanism and pathogenesis of IPF are still unclear, a possible pathological model has been proposed and involves micro-injuries of the alveolar epithelium. Following these injuries, fibroblasts migrate in the alveolar epithelium and start the deposition of fibrotic tissue. Moreover, fibroblast stimuli affect the alveolar type II cells, that in turn undergo EMT and acquire a fibroblast phenotype (Willis et al., 2005, 2006). A number of studies have identified pro-fibrotic pathways that are upregulated in IPF, such as the Wnt (Villar et al., 2011) and Hh (Cigna et al., 2012) pathways, but more recent studies have shown further possible mediators of EMT in pulmonary fibrosis. It is the case of Shp-1 (Src homology region 2 domain-containing phosphatase-1), that negatively regulates EMT via β-catenin inhibition (Chen et al., 2018), and of the NLRP3 (NOD-, LRR- and pyrin domain-containing protein 3) inflammasome, that may directly activate TGF-β1 signaling (Zheng R. et al., 2018). Moreover, it has been shown that the transcription factors SNAI (Snail family transcriptional repressor) 1 and 2 are involved in TFG-β1-induced EMT in alveolar epithelial type II cells (ATII). The overexpression of SNAIs in the nucleus of these cells induces EMT and contributes to the increased accumulation of myofibroblasts and of ECM in lungs of patients affected by IPF (Jayachandran et al., 2009).

Several possible strategies to counteract pulmonary fibrosis may be proposed for both PF and IPF. Regarding PF, a recent study has reported that inhibition of autophagy in alveolar epithelial cells promotes EMT and can contribute to fibrosis via an aberrant epithelial cells–fibroblasts crosstalk (Hill et al., 2019). In contrast with these observations, a previous study has reported that inhibition of mTOR, a central regulator of autophagy, by rapamycin, can revert the fibrotic phenotype in human lung fibroblasts.

In IPF, the mTOR pathway also enhances EMT in a TGF-β dependent manner (Tan et al., 2017). To support this mechanism, a recent study has demonstrated that in mice treated with bleomycin, which induces pulmonary fibrosis, the administration of rapamycin, by inhibiting mTOR, prevents EMT in epithelial alveolar cells (Han et al., 2018). On these bases, an upregulated autophagy results to be pro-fibrotic and EMT-inducing (Cho et al., 2015). Thus, autophagy inhibition can ameliorate the fibrotic disease. On the contrary other studies have shown that autophagy can be protective against pulmonary fibrosis development (Ryter and Choi, 2015; Gui et al., 2018), because the inhibition of autophagy pushes lung fibrotic cells towards EMT. This has been demonstrated by treating lung cells with leptin, a protein commonly correlated to obesity, which promotes the accumulation of α-SMA and collagen I in IPF and inhibits autophagy via the PI3K/Akt/mTOR pathway activation (Gui et al., 2018). Taken together these data suggest that targeting the autophagic process may represent a possible therapeutic strategy for pulmonary fibrosis, although a better understanding on the process’ involvement is needed.

Furthermore, the presence of macrophages expressing the activation marker MARCO (macrophage receptor with collagenous structure), has been discovered in lungs after their exposure to silica. Polyguanylic acid (PolyG) is a synthetic polynucleotide capable to inhibit MARCO and is capable to induce a decrease of the level of some EMT related proteins and transcription factors, such as vimentin and α-SMA in lung tissues. Thus, through the reduction of the EMT, PolyG may represent a feasible therapeutic option for the treatment of pulmonary fibrosis caused silicosis (Yang et al., 2019). In addition, a study performed both *in vitro* and *in vivo* has underlined a connection between EMT and hypoxia in lungs (Guo et al., 2015), thus the modulation of the HIF transcription factor, and the related EMT, could represent another approach for lung fibrosis therapy.

Recent discoveries have also shown that in chronic asthma inflammation there is an increased production of TGF-β1 that consequently mediates the EMT progression. Thus, inflammation of the airways could be considered a possible target in IPF treatment. In this regard, it has been shown that the administration of the Bacillus Calmette-Guerin (BCG) alleviates airway inflammation and remodeling through its ability to counteract the TGF-β1-induced EMT (Tian et al., 2017)

Regarding IPF, two agents (pirfenidone and nintedanib) have been approved by the Food and Drug Administration (United States) (Raghu and Selman, 2015). Nintedanib is a tyrosine kinase inhibitor targeting several growth factor receptors including: platelet derived growth factor (PDGF) alpha and beta, fibroblast growth factor (FGF) 1 and 2 and vascular endothelial growth factor (VEGF) 1, 2, 3, receptors. This drug seems to be effective mainly due to its high affinity for PDGFR (one of the EMT-inducing mediators) and FGFR (an important mediator of fibroblast proliferation), although, nintedanib is also able to interfere with myofibroblasts proliferation, differentiation and migration and with ECM deposition (Woodcock and Maher, 2015). Pirfenidone, on the other hand, is a non-peptide synthetic molecule able to inhibit TGF-β signaling by preventing the nuclear accumulation of Smad2 and 3 (Choi K. et al., 2012). Indeed, in a recent study carried out on a rat silicosis model treated with pirfenidone, it has been shown that the Smad2/3 expression and the TGF-β pathway signaling were downregulated (Guo et al., 2019). In addition, another study with pirfenidone versus placebo has shown that pirfenidone significantly reduces the progression of multiple disease associated with IPF (Nathan et al., 2019). Furthermore, it has been shown that rapamycin could potentiate the effects of pirfenidone and their combination has been shown to widen the inhibition of fibrogenic markers expression and prevents fibroblast migration in IPF (Molina-Molina et al., 2018). Pirfenidone also shows antioxidant and anti-inflammatory effects (probably through the inhibition of the pro-inflammatory cytokines synthesis associated with IPF). the two drugs are potentially useful also for the treatment of other fibrotic diseases. For instance, pirfenidone has been found to have a significant anti-fibrotic activity in kidney, where it reduced collagen accumulation and ECM expansion (Cho and Kopp, 2010) and a limited effect in liver, where the reversion of the fibrotic phenotype was observed only in a single animal model (Garcıìa et al., 2002). Nintedanib has been shown to have anti-fibrotic activity in systemic sclerosis, where the drug effectively reduced myofibroblasts differentiation and collagen deposition (Huang et al., 2016).

Other potentially interesting drugs for the treatment of IPF may be found in natural compounds. Diosgenin, extracted from the tubers of *Discorea.* Rats treated with diosgenin in bleomycin-induced IPF showed reduced EMT and TGF-β expression, together with reduced expression of pro-inflammatory mediators (Dinesh Babu et al., 2020). Another drug of natural origin that could be considered a candidate for the treatment of IPF is sulforane, a phytochemical found in some vegetables, that in alveolar lung cells inhibits TGF-β1-induced EMT and fibrosis (Kyung et al., 2018).

At the physiological level, a molecule that counteract fibrosis in both IF and IPF is decorin. This is a proteoglycan found in the extracellular matrix, where it is produced by fibroblasts, that is important for tissue homeostasis and collagen turnover. Both of these functions may be altered when decorin is degraded. In fact, elevated serum levels of degraded decorin are found in fibrotic lung diseases and, for this reason, this form of decorin has been proposed as a biomarker for lung fibrosis (Kehlet et al., 2017) while the wild-type form could be considered as a possible therapeutic agent because of its antifibrotic activity exerted by targeting TGF-β signaling. On these bases, decorin targeted therapy approaches have been proposed to treat fibrosis in wound healing (Järvinen, 2012; Järvinen and Ruoslahti, 2010, 2013), liver fibrosis (Baghy et al., 2012) and possibly for other tissue fibrosis (Järvinen, 2012) including pulmonary fibrosis.

Other strategies to counteract pulmonary fibrosis have been recently proposed, although in mouse experimental models. TGF-β1 is produced as a latent protein, thus it is necessary its activation by releasing it from the latent associated peptide (LAP) (Annes et al., 2003; Shi et al., 2011). This activation event requires a decrease in extracellular pH and degradation of the LAP. It has been shown that production of lactic acid by fibroblasts may mediate TGF-β1 activation. The enzyme responsible for lactic acid production is lactic acid dehydrogenase-A (LDH-A), that is found elevated in lung tissue from patients with IPF (Kottmann et al., 2012). Thus, a possible therapeutic approach for IPF would be the inhibition of LDH-A in order to inhibit TGF-β1 activation and its induced myofibroblasts differentiation. A study has shown that gossypol, a small molecule inhibitor of LDH-A isolated from cottonseed oil, is effective at inhibiting and treating experimental pulmonary fibrosis, thus LDH-A may be considered a potential target for pulmonary fibrosis therapy (Judge et al., 2018).

**FIGURE 6:**
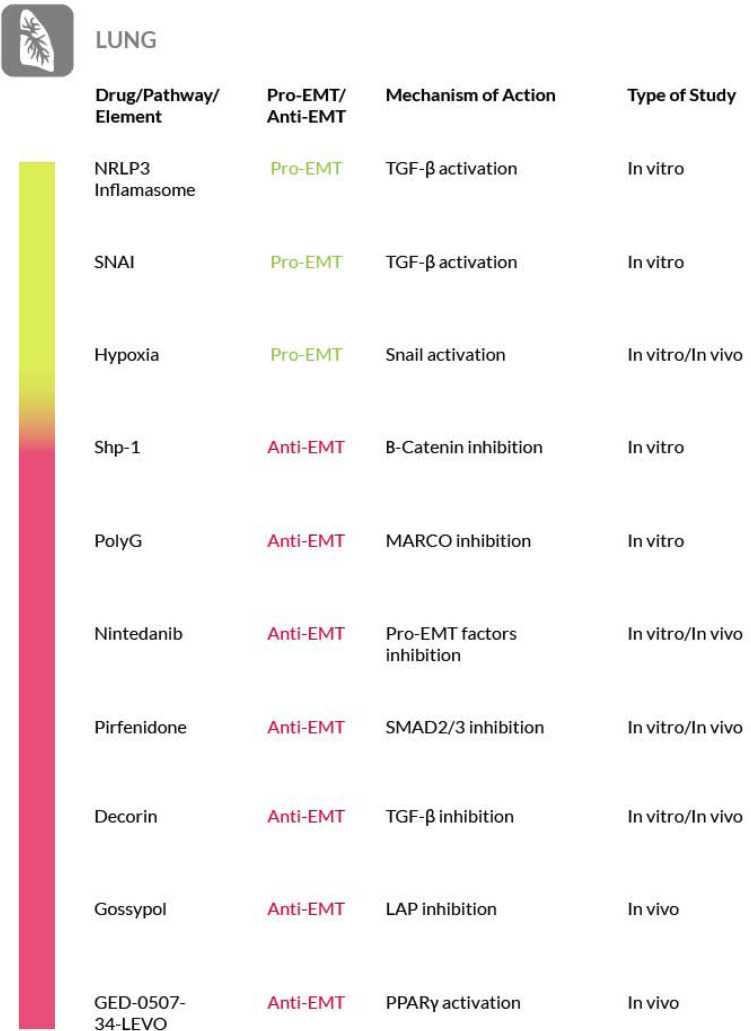
Most relevant molecular mechanisms and possible drugs for the treatment of EMT in pulmonary fibrosis.

Furthermore, a study on a murine model of pulmonary fibrosis has recently been conducted to evaluate the safety and efficacy of an original PPARγ modulator called GED-0507-34-Levo (GED-0507), in halting or even reversing pulmonary fibrosis induced by bleomycin. Preventive and curative administration of GED-0507 improved both loss of body weight and extension of lung fibrotic lesions. When given in preventive mode GED-0507 was also able to abrogate mortality and the histological score of fibrosis (Speca et al., 2020) (Figure 6).

### Intestinal Fibrosis

In the intestine, fibrosis is often a complication of a wide range of enteropathies. However, intestinal fibrosis is most often associated with the inflammatory bowel disease (IBD) as a consequence of the persistent inflammatory stimuli typical of both ulcerative colitis (UC) and Crohn’s disease (CD), with the latter showing a wider fibrotic reaction (Latella et al., 2014). An important trait of intestinal fibrosis is that it becomes independent from the inflammatory stimuli in its advanced stage. This feature is not shared with the fibrosis of other organs and underlines the fact that the fibrotic process is mediated by complex cellular and molecular mechanisms that may act differently, and even in the opposite way, in different organs. In fact, some well-known anti-fibrotic mediators in organs such as lung and liver, are pro-fibrotic in the intestine. It is the case of IFN-γ, that in combination with other pro-inflammatory cytokines, such as IL-1α and TNF-α, greatly increases TGF-β production in intestinal epithelial cells. The highest levels of TGF-β have been observed in experimental models when IL-1α, TNF-α and IFN-γ were used together, showing an additive pro-fibrotic effect (Drygiannakis et al., 2013). In intestinal fibrosis, EMT is the main source of myofibroblasts (Burke et al., 2010; Davids et al., 2010; Vetuschi et al., 2015). In fact, the typical hallmarks of EMT have been found expressed in both UC and CD (Scharl et al., 2015), and the EMT has been recognized as a pathological mechanism behind intestinal fibrosis (Medina et al., 2011). In particular, it has been recently discovered that a specific factor, the parathyroid hormone–like hormone (PTHLH), induces EMT in intestinal epithelial cells of CD patients, by activating the PKA-Runx2 pathway (protein kinase A - runt-related transcription factor 2) which therefore may be considered as a possible therapeutic target (He et al., 2018). Moreover, a recent study in cells derived from the intestinal mucosa of CD patients has reported that IL-17A activity is involved in the fibrosis-associated EMT through an increased expression of mesenchymal markers, such as vimentin and Snail, underlining the EMT activation (Zhang H. et al., 2018). As additional evidences for the EMT involvement in intestinal fibrosis, several studies have reported that the aberrant signaling of TGF-β and of its crosstalk pathways (mTOR or Hh) lead to EMT, Johnson et al. (2013) and inhibition of EMT reverts the fibrotic phenotype (Flier et al., 2010; Xu et al., 2017). Other signaling pathways and cellular processes that are involved in other organ fibrosis may also have a potential role in intestinal fibrosis by affecting the TGF-β signaling and the EMT. A recent *in vivo* study has underlined a possible role for the Wnt pathway (Di Gregorio et al., 2017). I, and a mouse model of colitis showed that, during the epithelial to myofibroblast transition, there is an increase of vimentin instead of E-cadherin, a process mediated by the cooperation of the Wnt and Notch pathways (Lovisa et al., 2019).

Furthermore, in intestinal fibrosis, PPAR-γ activation, i.e., by synthetic ligands, has been shown to restore the epithelial phenotype and the stability of the E-cadherin/β-catenin complex, by acting on Smad and non-Smad mediated EMT, suggesting that this mechanism may be exploited to reverse EMT (Flier et al., 2010; Di Gregorio et al., 2017). PPAR-γ activators may reverse the fibrotic state and some of these activators are giving good results, at least in experimental setups (Kulkarni et al., 2011; Xu et al., 2017; Di Gregorio et al., 2017). A drug with these properties is GED-0507-34 Levo (GED), an aminophenyl-alpha-methoxypropionic acid (Speca et al., 2016, 2020).

There are also molecules from natural sources that could be effective against intestinal fibrosis. One of them is curcumin, the main curcuminoid of turmeric (Curcuma longa), which activates PPAR-γ by promoting its nuclear translocation and expression (Xu et al., 2017). Moreover, administration of apple pectin, a soluble dietary fiber found naturally in apples, in a mouse model of radiation-induced intestinal EMT and fibrosis, has been shown to protect against EMT (Yang et al., 2017a). Furthermore, silibinin, a polyphenolic flavonoid derived from the seeds and fruits of the medicinal plant Silybum marianum (milk thistle), has been proposed for the treatment of irradiation-induced intestinal EMT, in patients affected by abdominal tumors who undergo pelvic radiotherapy. In fact, high TGF-β1 levels and intestinal fibrosis are induced by radiations. In mice, silibinin inhibits intestinal fibrosis by inactivating Smad2/3 phosphorylation and by reversing EMT (Kim et al., 2017).

Another possibility to counteract the development of intestinal fibrosis consists in the use of microvesicles containing miR-200b. These microvescicles have been used to transduce intestinal epithelial cells and colon tissues and, in both cases, miR-200b has been shown to be effective in preventing EMT and in alleviating fibrosis, by reducing the expression of EMT-related proteins such as ZEB1 and ZEB2, namely the targets of miR-200b (Yang et al., 2017b). Furthermore, Mesenchymal Stromal Cells (MSCs) have been shown to possess anti-fibrogenic traits both in prophylaxis and therapy of CD-associated intestinal fibrosis induced by 2,4,5-trinitrobenzene sulphonic acid (TNBS). MSCs cause the inactivation of the TGF-β/Smad signaling pathway with a negative modulation of the inflammatory and EMT responses (Lian et al., 2018). Further potential targets for an anti-fibrotic therapy are the Rho kinases (ROCKs). These are serine/threonine kinases, regulated by the small GTPase Rho, that are involved in EMT and autophagy (Mleczak et al., 2013; Huang et al., 2015). ROCKs are expressed in diverse cell types of the intestinal tract and are activated in fibrotic tissues. ROCK inhibition has been tested as a treatment for pulmonary fibrosis but it has shown important side effects by reaching the systemic circulation (Olson, 2008). More recently a ROCK inhibitor called AMA0825, has been tested for intestinal fibrosis also because of its long retention time in the gut and because it is quickly degraded by esterases in the systemic circulation, thus assuring localized drug effects and eliminating the side effects deriving from systemic circulation. Treatment with AMA0825 inhibits myofibroblast accumulation, expression of pro-fibrotic factors, and accumulation of fibrotic tissue in experimental models of fibrosis. Furthermore, ROCK inhibition has been shown to reverse established fibrosis in a chronic DSS model. The AMA0825 molecular mechanism of action includes a reduction of TGFβ1-induced activation of myocardin-related transcription factor (MRTF) and p38 mitogen-activated protein kinase (MAPK), and a potentiated autophagy. Thus a local ROCK inhibition may represent a possible mean to counteract fibrosis in the intestine (Holvoet et al., 2017).

Recently, in order to better understand the mechanisms favoring the progression of EMT and fibrosis of the bowel, a study has also investigated the involvement of the Zinc Finger Protein 281 (ZNF281), another EMT-inducing transcription factor. This molecule is involved in various diseases, such as cancer stemness, but it also plays an important role in fibrosis. In fact, in ZNF281 knock out-mice, the level of EMT inducers (IL-1β, IL-17, IL-23) is decreased, suggesting that silencing of ZFN281 may represent a possible option for bowel fibrosis therapy (Pierdomenico et al., 2018).

**FIGURE 7:**
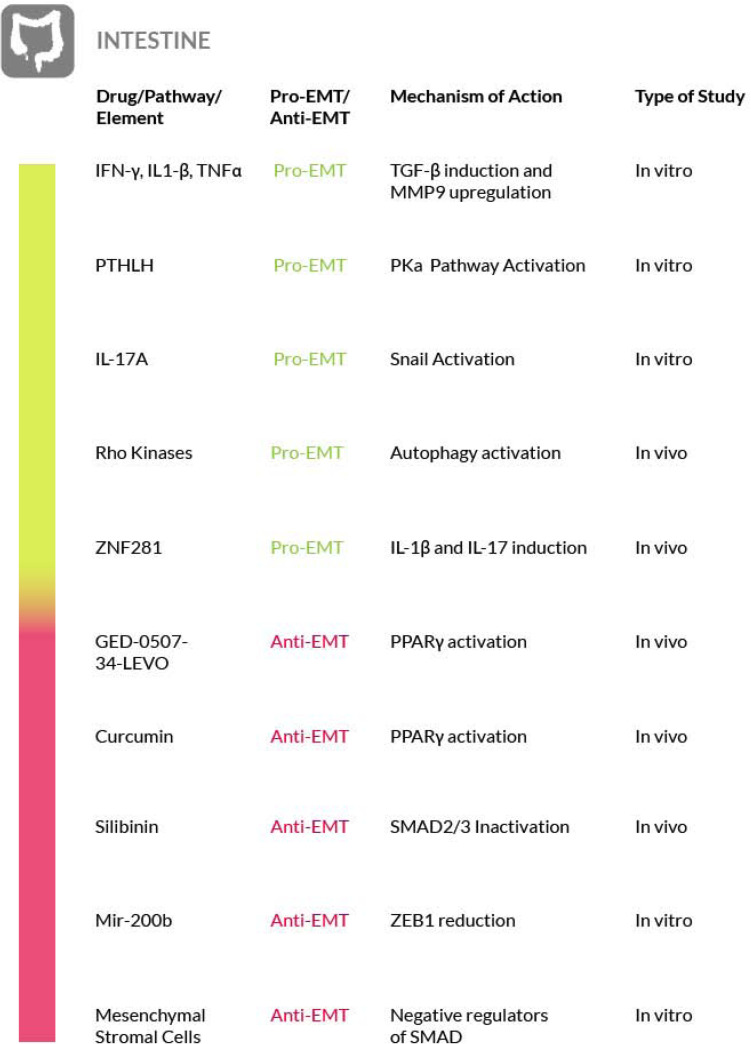
Most relevant molecular mechanisms and possible drugs for the treatment of EMT in intestinal fibrosis.

Furthermore, in intestinal fibrosis deserves attention the possible pathological role of the microbiome. In fact, studies performed in mice have shown that the microbiome may be responsible for the perpetuation of intestinal fibrosis, while in mice deprived of intestinal microbes, the inflammation and the resulting fibrosis is attenuated. This has been attributed to the microbial metabolic products, which are able to pass the gut barrier and to stimulate the canonical TGF-β pathway and so the EMT (Rieder, 2013; Matarazzo et al., 2018). However, further studies are needed to substantiate the role of the microbiome in intestinal fibrosis and consequently its possible targeting (Figure 7).

### Ocular Fibrosis

Fibrosis is at the basis of several diseases affecting different districts and cell types of the eye,. In all these cases, the fibrotic reaction leads to a loss of function of the eye and ultimately to blindness. Two common features of these fibrotic diseases are the dysregulation of the eye normal wound healing, that leads to myofibroblasts proliferation, and the enhanced canonical TGF-β signaling (Shu and Lovicu, 2017). The canonical TGF-β/Smad pathway plays a major role in ocular fibrosis, inducing EMT. Flanders (2004) and Singh et al. (2012). A study carried out on murine corneal progenitor cells, showed the induction of irreversible EMT following cell senescence induced by TGF-β through the Smad pathway (Kawakita et al., 2013). More recently, in a murine model, it has been demonstrated the role of galectin-1 (LGALS1) as a promoter of the TGF-β receptor signaling, and the overexpression of LGALS1 results in EMT-induced subretinal fibrosis in patients affected by age-related macular degeneration (AMD) (Wu D. et al., 2019).

The key role played by EMT in ocular fibrosis is underlined by the observation that while in other organs the myofibroblasts could derive from both EMT and fibroblast to myofibroblast transformation, in the eyes, especially in crystalline lens, myofibroblasts originate only from EMT. Upon direct TGF-β stimuli, normal cells of the eye such as keratocytes, lens epithelial cells, trabecular meshwork cells, or retinal pigment epithelial cells, undergo EMT, acquire myofibroblast phenotype and enhance ECM deposition (Saika et al., 2008). Furthermore, in lens epithelial cells, the Myocardin-related transcription factor A (MRTF-A), an actin-binding protein (ABP), could affect TFG-β-induced EMT, resulting in the enhanced expression of α-SMA, a marker typically expressed by myofibroblasts (Korol et al., 2016). Other mechanisms involved in EMT activation in the eye tissues have also been reported; for example, in trabecular meshwork cells, induction of paxillin, a protein codified by the PAX gene and involved in cellular-ECM adhesion, leads to EMT activation, while in other cell types, such as retinal pigment epithelial cells (RPE), the EMT is mediated by TNF-α and the hyaluronan receptor CD44 (Takahashi, 2016).

For therapeutic purposes, the most studied approach in ocular fibrosis consists in targeting EMT via TGF-β signaling inhibition. In this regard, pirfenidone containing nanoparticles have been shown to be effective by decreasing collagen synthesis and preventing myofibroblast formation (Chowdhury et al., 2013). However, the antagonism of TGF-β signaling, even considering the good results obtained with pirfenidone or with BMP7 induction or the preliminary results obtained with Smad7 gene therapy (Fuchshofer et al., 2007, 2009), may present possible drawbacks resulting from the involvement of the TGF-β signaling in the eye normal wound healing (especially in the cornea), and from redundant signaling that may bypass TGF-β signaling inhibition. For instance, recent studies have reported the enhancement of the Wnt signaling in lens epithelial cells as another pro-EMT pathway (Chong et al., 2009; Lovicu et al., 2016; Wei et al., 2017; Shu and Lovicu, 2017). According to these studies, Wnt signaling is required for TGF-β induced EMT in ocular fibrosis, thus, it may represent an additional therapeutic target for ocular fibrosis, although more studies are needed to support this possibility. The search for effective ocular fibrosis therapies is very important because there are fibrotic diseases of the eye that do not respond effectively to the currently available therapies. Among them, macular fibrosis is particularly important because it represents the final pathological step of neovascular age-related macular degeneration (nAMD), the most common cause of blindness among aged people (Bressler, 2004). The most efficient therapy for nAMD is represented by the intravitreal injection of anti-VEGF drugs. Patients treated in the early steps of nAMD unlikely develop macular fibrosis. Unfortunately, about 30% of the patients do not respond to anti-VEGF therapy, also due to macular degeneration (Little et al., 2018). Thus, an effective treatment, alternative or additional to anti-VEGF drugs, to prevent or to treat macular fibrosis, would be very welcome for these patients and the interference with the TGF-β and Wnt pathways could be explored for this purpose. Recently, we have shown that this is not the only possible approach to counteract EMT in the eyes. In fact, we have reported that cerium nanoparticles (nanoceria) exert an anti-EMT activity in the RPE, in a light-damage rat model of nAMD, by modulating autophagy and cell survival of the RPE cells (Tisi et al., 2020).

**FIGURE 8:**
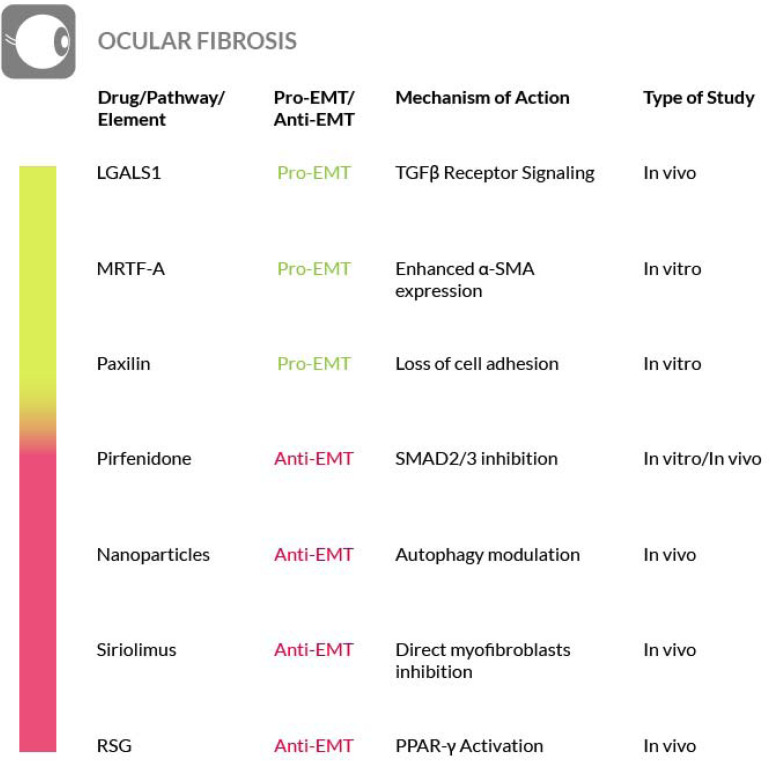
Most relevant molecular mechanisms and possible drugs for the treatment of EMT in ocular fibrosis.

Recent studies have shown an improvement in patients affected by fibrotic sequelae of Graves orbitopathy, or thyroid eye disease (TED), and the reversal of extraocular muscle fibrosis if treated with sirolimus (rapamycin). This drug has been shown to exert its anti-fibrotic activity by acting on myofibroblasts, the primary ECM-secreting cells during wound healing and fibrosis (Roos and Murthy, 2019). Furthermore, glaucoma filtration surgery (GFS) is the most commonly used surgical procedure for glaucoma treatment but postoperative fibrosis is still a major threat for the success of this treatment. Sirolimus has a known antifibrotic activity on vascular endothelium and cultured fibroblasts, but it also decreases the intraocular pressure (IOP) and determines a milder fibrosis in treated eyes, compared to control eyes. On these bases, although further studies are needed, rapamycin is a promising therapeutic approach to also control postoperative fibrosis in glaucoma patients undergoing filtration surgery (Yan et al., 2011; Cinik et al., 2016). Furthermore, in an *in vivo* rabbit model of GFS, has also been evaluated the potential antifibrotic effect of rosiglitazone (RSG), a PPARγ-selective agonist, on subconjunctival fibrosis. It has been shown that RSG treatment prevents subconjunctival fibrosis after GFS through the inhibition of profibrotic gene expression by a mechanism that promotes autophagy in local fibroblasts. Thus, the use of RSG may represent a novel antifibrotic approach with the potential to improve the success rate of GFS (Zhang et al., 2019) (Figure 8).

### Scleroderma

Scleroderma, or systemic sclerosis (SSc), is a complex and heterogeneous autoimmune disease that includes the development of fibrosis. In SSc the fibrotic process affects the skin and other organs at once and the TGF-β and Wnt/β-catenin signaling pathways play a crucial role in this process. These pathways are regulated at the post-translational level by modification of ubiquitination and mutations of ubiquitination enzyme genes have been found in scleroderma patients (Long et al., 2018). Furthermore, a role for mast cells, innate immune cells commonly known to play a key role in anaphylaxis (Toniato et al., 2017; Maurer and Pucillo, 2018), has also been found in the initiation of the sclerotic process (Bagnato et al., 2016; Bradding and Pejler, 2018). At the early stages of scleroderma, when mast cells accumulate in a specific tissue such as the dermis, forming an inflammatory infiltrate, they start to interact with fibroblasts (Willenborg et al., 2014; Gurung et al., 2019). The strong influence of the increased dermal mast cell population on fibroblasts leads to the activation of the latter, thus promoting the onset of extracellular fibrogenesis (Yamamoto et al., 2000; Sköld et al., 2001; Gailit et al., 2001; Gruber, 2003). The number of mast cells is elevated in all fibrotic conditions and their density within a given tissue may be affected by their release of granules containing numerous fibroblast-activating factors, mitogens and cytokines (Overed-Sayer et al., 2013; Mukai et al., 2018). Among the mast cell–derived secretory products there is also tryptase, a protease that serves as a marker of mast-cell activation (Payne and Kam, 2004). This enzyme exerts a fibrogenic activity, by triggering collagen production by the fibroblasts (Akers et al., 2000; Levi-Schaffer and Piliponsky, 2003). Although its role, as a mediator of the fibrotic process, has been analyzed in a few studies (Kondo et al., 2001; Bagher et al., 2018), further studies are required to increase the knowledge on its functions and to explore the possibility of using inhibitors targeting the enzyme for an antifibrotic therapeutic intervention. Furthermore, the contribution of plasminogen activator inhibitor-1 (PAI-1) to fibrosis progression through the activation of mast cells has recently emerged. In particular, high PAI-1 levels have been found in skin fibrosis where PAI-1 promotes mast cells-fibroblasts contact and may act as a molecule that conveys mast cells to the tissue site (Pincha et al., 2018). Nevertheless, there are contrasting evidences regarding the true role of mast cells in fibrosis that must be taken in consideration in order to develop effective strategy for their targeting (Bradding and Pejler, 2018). Other immune cells that play a key role in the pathogenesis of the sclerosing disorder are the activated macrophages. In fact, failure to resolve macrophage activation can lead to chronic inflammation and fibrosis. For a detailed discussion of the therapeutic interventions that could be implemented by targeting activated macrophages in SSc, we refer the reader to a recent review (Toledo and Pioli, 2019).

The correlation between scleroderma and cutaneous EMT is supported also by other evidences. The transition process induced by TGF-β in primary keratinocytes is enhanced by TNF-α. Both of them are important EMT mediators and are overexpressed in scleroderma and in combination they contribute to the activation of the Smad-mediated signaling pathway, that consequently could be inhibited to reverse the EMT process (O’Kane et al., 2014). Moreover, the findings of a study focused on morphea, a form of localized scleroderma, suggest the involvement of EMT also in this type of skin disease, on the basis of the expression level of fibrosis (TGF-β1, αSMA and fibronectin) and EMT (Snail1 and E-cadherin) markers, although only the EMT markers were found reduced in this localized scleroderma (Takahashi et al., 2013). Furthermore, in scleroderma has been shown that epithelial cells could be activated in a TGF-β dependent manner thus acquiring partial mesenchymal features. The EMT model was reproduced in a SSc keratinocyte cell line, under TGF-β stimulus, and a partial EMT-like process, without E-cadherin loss, was observed (Nikitorowicz-Buniak et al., 2015). Hence, although a better understanding of skin and systemic fibrosis mechanisms is needed in order to identify potential therapeutic targets, the mechanisms regulating the EMT process may represent a source of such targets. Unfortunately, to date an effective therapy for treating patients with SSc is not available. To this purpose, several studies have been conducted on mast cells. Degranulation of mast cells, mediated by Toll Like Receptor (TLR) or NOD Like Receptor (NLR) activation, releases a large amount and variety of molecules, including inflammatory cytokines and pro-fibrotic factors. It has Blockade of mast cells activation, through inhibition of the tyrosine kinase receptor c-kit by masitinib, may be effective for scleroderma therapy (Hügle, 2014). Another experimental approach to restrain mast cells degranulation and to block the consequent fibrotic process has been proposed by acting on the innate and adaptive immune response. IL-37 is one of the molecules involved in immune response inhibition and it has been proven that it can stop the TLR signaling pathway and consequently prevent the onset of fibrosis mediated by mast cells activation (Conti et al., 2018). Another drug that can block the onset of fibrosis or its progression, is ketotifen, a histamine release inhibitor. It plays an important role in the stabilization of mast cells at the early stages of wound healing and it reduces the onset of fibrosis without altering wound healing. This is just a clue of the potential use of this drug as a therapeutic agent against fibrosis in systemic sclerosis (Gallant-Behm et al., 2008).

In addition, the EMT has been proposed as a source of myofibroblast also in SSc (Gillespie et al., 2011; Nikitorowicz-Buniak et al., 2015) and EMT-related pathways, such as the Wnt or TGF-β induced pathways, have been shown to be involved in scleroderma (Lee et al., 2020). Furthermore, the Endo-MT process has also been found in the target organs of SSc (Thuan et al., 2018), where it is activated by the inflammatory leukocytes recruited in the early stage of the disease. EndoMT, then, could contribute to exacerbate the pro-fibrotic TGF-β signaling in SSc. On these bases, some of the anti-EMT strategies that we have highlighted earlier in the paper could be valuable also for the SSc. In particular, the process of Endo-MT can represent a therapeutic target in the early stages of the disease (Mendoza et al., 2016).

**FIGURE 9:**
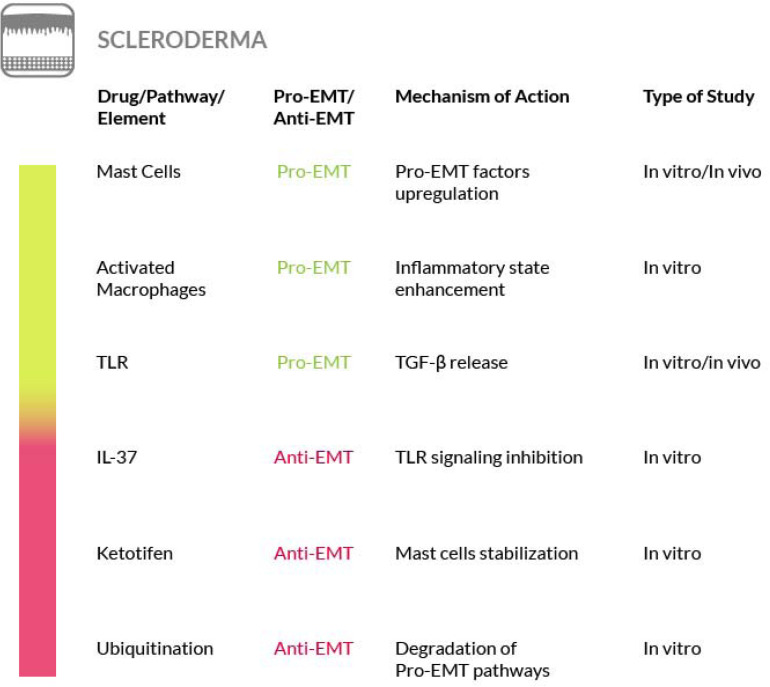
Most relevant molecular mechanisms and possible drugs for the treatment of EMT in scleroderma.

Another option that may be considered for the treatment of SSc is by interfering with the ubiquitination of the main components of the TGF-β and Wnt/β-catenin pathways. In fact, both pathways are regulated by ubiquitination and recently it has been reported that ubiquitination is dysregulated in patients with SSc. Thus, the understanding of the mechanisms underlying the ubiquitination alteration in this disease, may constitute the basis for developing a new treatment (Long et al., 2018) (Figure 9).

### Adipose Tissue Fibrosis

Adipose tissue fibrosis is frequently associated with obesity: the adipose tissue expands by hyperplasia and hypertrophy and the combination of these two processes leads to an inadequate tissue oxygen supply and the subsequent development of hypoxia. In this condition HIF-1α is activated and induces adipose tissue fibrosis. In addition, the insufficient oxygen content induces the macrophages of the adipose tissue to release TGF-β, PDGF, cytokines and chemokines, that attract inflammatory cells and fibroblasts, laying the foundations of the inflammatory process. In these conditions, there is also a depletion of PPAR-γ levels in fat cells, causing the inhibition of pre-adipocyte differentiation. Thus, triglycerides surplus is not stocked in lipid droplets of fat cells but its storage occurs ectopically. The consequent stress condition, associated with hypoxia, induces migration of immune cells and stimulates inflammation, thus initiating and exacerbating adipose tissue fibrosis, through the increased production of collagen. Among the immune cells, macrophages and mast cells play a key role in adipose tissue fibrosis. In fact, obesity is a condition in which the number of resident macrophages, especially the pro-inflammatory M1 type, results to be augmented in fat tissue (Buechler et al., 2015). On the other hand, it has been reported that in the fibrotic areas of the adipose tissue tend to localize the M2 macrophages that release TGF-β and exert an anti-inflammatory function (Spencer et al., 2010, 2011). Furthermore, the notion that adipose tissue fibrosis is driven by inflammation has been disputed by studies on mice fed with a high fat diet where has been shown that the fibrotic streaks in the adipose tissue appear before the onset of inflammation (Marcelin et al., 2017; Halberg et al., 2009). Thus, the role of polarized macrophages in adipose tissue fibrosis has yet to be clarified. In addition, it has been observed that inflammation of the adipose tissue of obese subjects may also be associated with hyperoxia (Goossens et al., 2011; Goossens and Blaak, 2015). T In addition, it must be underlined that a correlation between adipose tissue hypoxia and fibrosis has been found in rodents but not in humans, although adipocyte hypoxia has been associated with EMT promotion in breast cancer (Yao-Borengasser et al., 2015). Furthermore, together with preadipocytes and adipocytes, cells of the immune system, such as the mast cells, can also accumulate in fat tissue and synthesize a high amount of collagen, thus promoting the fibrogenic progression (Khan et al., 2009; Rutkowski et al., 2015; Buechler et al., 2015; Muir et al., 2016). In addition, a connection between adipose tissue fibrosis, obesity, and the obesity-related metabolic diseases, has been found (Muir et al., 2016). In fact, the development of adipose tissue fibrosis is also associated with metabolic alterations, such as type 2 diabetes (Hasegawa et al., 2018). Thus, targeting adipose tissue fibrosis could involve metabolic alterations in obese subjects. On this regard, it must be pointed out that adipose tissue fibrosis may exert a protective function against the insurgence of metabolic alterations in obese subjects (Muir et al., 2016; Datta et al., 2018).

**FIGURE 10:**
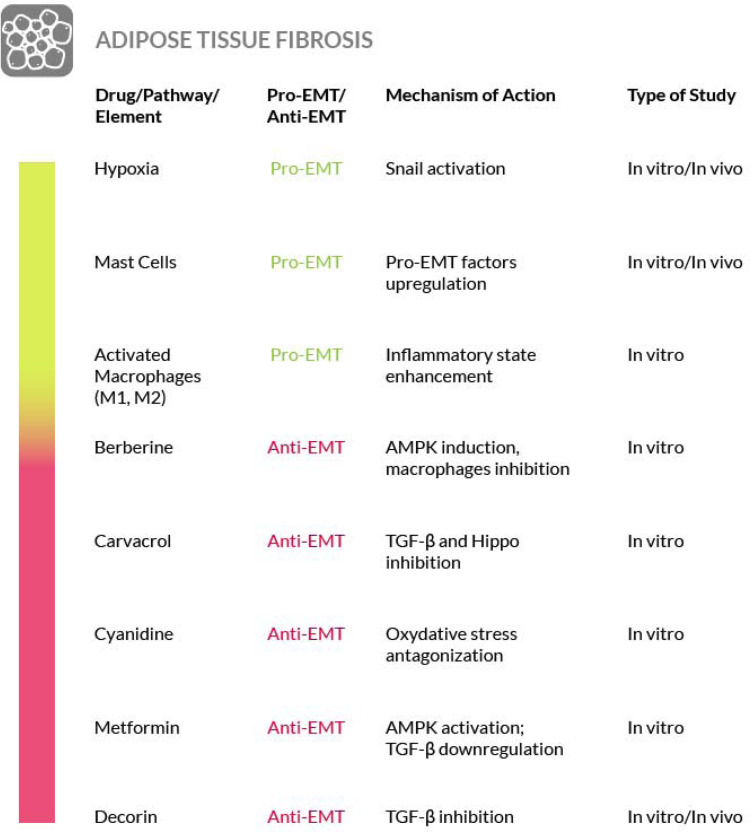
Most relevant molecular mechanisms and possible drugs for the treatment of EMT in adipose tissue fibrosis.

A recent study has shown that the alkaloid berberine, a natural compound found in several plants, by inducing AMPK activity and by inhibiting TGF-β1/Smad3 signaling, attenuated infiltration and polarization of adipose tissue macrophages, attenuated collagen deposition and alleviated adipose tissue fibrosis (Wang L. et al., 2018). Other natural anti-adipogenic substances, like cyanidin and carvacrol, Pompei et al. (2012) and Spalletta et al. (2018) may also restrain adipose tissue fibrosis, as recently seen for liver fibrosis. In particular cyanidin has been proven to be effective in antagonizing the oxidative stress, the inflammatory response and HSC activation; while carvacrol has been found to be particularly successful in inhibiting the TGF-β signaling pathway, by downregulating not only the TGF-β levels but also the protein levels of the related Hippo cascade mediators such as TAZ and YAP (Cho et al., 2014; Jiang et al., 2015; Hussein et al., 2017; Mohseni et al., 2019). Blockade of these signaling pathways could inhibit EMT and ameliorate fat fibrosis, as well as the associated metabolic dysfunctions such as insulin resistance. In fact, in another recent study has been shown that metformin could mitigate fibrosis and glucose intolerance induced by doxorubicin, a cancer chemotherapy drug, in subcutaneous adipose tissue. Doxorubicin induces severe damage of the adipose tissue by interfering with AMPK and PPAR-γ signaling. Metformin, by activating AMPK, has been shown to prevent this side effect thus it is proposed that, with a similar mechanism, it could be effective also against adipose tissue fibrosis (Biondo et al., 2018). On the other hand, administration of PPARγ agonists or adiponectin both reduce adipose tissue fibrosis through the reduction of collagen levels (Khan et al., 2009). Furthermore, recently, it has been reported that the small leucine-rich proteoglycans lumican and decorin have an important role in EMT occurring during fibrosis in obesity, but with contrasting final effects. In fact, while lumican exerts a stimulatory effect on EMT, decorin exerts an inhibitory effect by contrasting TGF-β activity (Pierleoni et al., 1998; Alvarez-Llamas et al., 2007; Molina et al., 2009). The reduced TGF-β activity is reflected on a reduced synthesis and an increased degradation of ECM proteins such as collagens. In particular, collagen VI is highly expressed in adipose tissue and plays a key role in the fibrosis associated with obesity, thus its degradation, promoted by decorin, may counteract the development of adipose tissue fibrosis and so it could be leveraged for therapeutic purposes (Kuo et al., 1997) (Figure 10).

### Cardiac Fibrosis

In cardiac or myocardial fibrosis, a substantial accumulation of extracellular matrix proteins occurs in the cardiac interstitium (interstitial fibrosis). The matrix remodeling and fibrotic response can follow myocardial infarction, during which acute death of cardiomyocytes results in an intense inflammatory reaction that contributes to the replacement of the injured tissue with scar tissue (replacement fibrosis) (Disertori et al., 2017). When the homeostatic balance between the proper synthesis and degradation of matrix proteins is impaired, structural and functional changes of the tissue take place thus allowing the development of fibrosis (Frangogiannis, 2019). In other cases, cardiac fibrosis is associated with aging and/or cardiac and metabolic pathological conditions, in absence of cardiomyocytes death. Several cells, such as immune cells (macrophages, monocytes, mast cells and lymphocytes), cardiomyocytes and vascular cells, secrete fibrogenic mediators. Among them there are chemokines, cytokines, TGF-β, TNF-α, PDGF, Endothelin (ET)-1, FGF, ROS, the renin-angiotensin-aldosterone system (RAAS) and specific proteases that regulate the fibrotic process (Frieler and Mortensen, 2015; Strassheim et al., 2019; Psarras et al., 2019; Okyere and Tilley, 2020).

Mast cells, in particular, contribute to cardiac fibrosis by releasing the content of their granules, including the enzymes tryptase and chymase, pro-inflammatory cytokines and mitogens that act as fibrogenic mediators. All of these factors contribute to fibroblast proliferation and to collagen deposition. We refer you to a review for a detailed discussion on the role of mast cells in the pathophysiology of cardiac fibrosis (Levick et al., 2011).

Under the influence of some of the above-mentioned mediators, cardiac fibroblasts transdifferentiate in myofibroblasts, acquiring a phenotype similar to that of smooth muscle cells. Myofibroblast transdifferentiation converts fibroblasts in secretory cells that serve as further sources of fibrogenic factors. This may be considered the main mechanism responsible for cardiac fibrosis (Kong et al., 2014). Furthermore, fibroblasts can also originate from endothelial cells under TGF-β stimuli. In fact, endothelial cells undergo EndoMT that give rise to the progression of cardiac fibrosis (Zeisberg E. M. et al., 2007). In addition to EndoMT, the EMT process may also be involved in cardiac fibrosis because, during development, TGF-β-driven EMT is also responsible for cardiac fibroblasts formation (Travers et al., 2016). This process appears to be regulated by the Hippo pathway as well, as seen in a recent *in vitro* study (Aharonov et al., 2020). Nevertheless, further studies are required to understand if the deregulation of this process may contribute to cardiac fibrosis as a source of myofibroblasts (Moore-Morris et al., 2014). To date, the therapeutic strategies for cardiac fibrosis have been proposed either to inhibit the profibrotic stimuli or to stimulate antifibrotic pathways. Among them, a possible strategy may rely on the inhibition of the non-canonical TGF-β signaling pathway. In fact, inhibition of the TAK1 signaling (that is associated with EMT, as we have seen earlier in this paper) determines a lower ECM production in the heart, and so decreases cardiac fibrosis (Ono et al., 2003; Bhandary et al., 2018). Thus, targeting both TGF-β and TAK1 signaling pathways may enhance the antifibrotic activity and represent a possible combination therapy for cardiac fibrosis. TGF-β signaling is physiologically repressed by the c-Ski family of proteins through interactions with the Smad proteins (Akiyoshi et al., 1999; Luo et al., 1999; Stroschein et al., 1999). Nevertheless, c-Ski, may be also associated with TGF-β-induced EMT (Suzuki et al., 2004) and cardiac fibrosis (Cunnington et al., 2009; Dixon et al., 2008). In fact, in cardiac muscle cells, c-Ski expression is downregulated by TGF-β and c-Ski gene silencing aggravates the fibrogenic transformation process stimulated by TGF-β. Conversely, c-Ski upregulation has the effect of restoring E-cadherin expression and of suppressing α-SMA and fibronectin expression (Ling et al., 2019). Thus, the modulation of c-Ski expression could represent a means to counteract EMT in cardiac fibrosis.

Furthermore, the role of the known EMT regulator Snail, has been investigated in cardiac fibrosis. In fact, after myocardial infarction, in mice, Snail1 has been found co-expressed and increased together with periostin, a cardiac fibrosis marker. This finding may involve Snail1 among the prospective targets also in cardiac fibrosis therapy (Liu et al., 2012). Another possibility to counteract cardiac fibrosis after myocardial infarction, is by modulating the G protein coupled receptors (GPCRs). These proteins are important mediators of the physiological EMT during heart development thus they may be targeted to reprogram EMT (Nebigil and Désaubry, 2019).

As we have seen earlier in this paper, several miRNAs have been found closely related to fibrosis. Among them, the role of miR-199a in the heart disease has been evaluated in an experimental model of myocardial fibrosis induced by isoproterenol. After isoproterenol treatment, the level of microRNA-199a was found increased and could regulate rat myocardial fibrosis by targeting secreted frizzled-related protein 5 (SFRP5). Thus, an inhibitor of this microRNA could be effective to reduce the cardiac fibroblast to myofibroblast transformation in cardiac fibrosis (Chen et al., 2019). The miRNAs could be also effective themselves as therapeutic agents. For instance, miR-378 is secreted from cardiomyocytes in response to mechanical stress and acts as an inhibitor of cardiac fibrosis through a paracrine mechanism that mediates the inhibition of p38 MAPK phosphorylation in cardiac fibroblasts (Yuan et al., 2018) as also shown by the administration of microvesicles containing the miR-378, so mir-378 could be studied as a mean to attenuate myocardial fibrosis (Liu W. et al., 2019). Other miRNAs implicated in cardiac fibrosis and more related to the EMT are represented by the members of the miR-133 family (Li N. et al., 2018). These miRNAs are abundantly expressed in the heart and miR-133a has been found to directly represse the expression of Snail1 (Li et al., 2010; Muraoka et al., 2014). Furthermore, miR-133a mimics or miR-133a overexpression have been found to significantly decrease cardiac fibrosis in chronic heart failure rat models (Sang et al., 2015) and overexpression of mir-133a can reduce myocardial fibrosis in adult mouse hearts (Matkovich et al., 2010). Thus, the miR-133 family may be considered a good source of candidates for developing a therapy for cardiac fibrosis by interfering with EMT.

**FIGURE 11:**
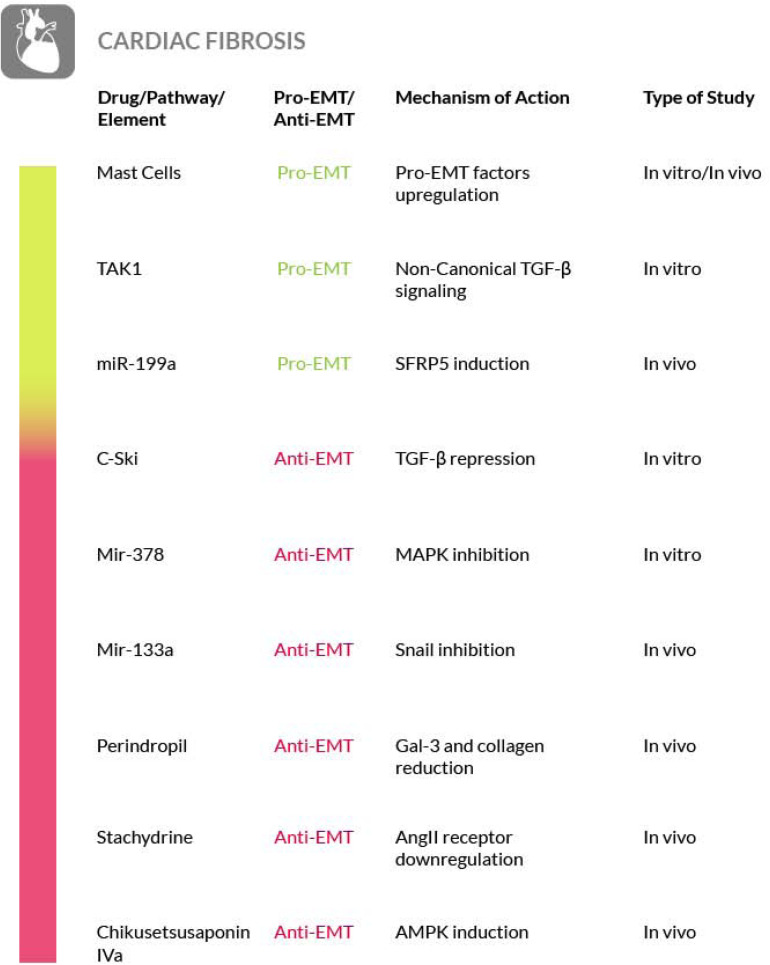
Most relevant molecular mechanisms and possible drugs for the treatment of EMT in cardiac fibrosis.

In addition, some natural compounds have also been evaluated for their effectiveness in improving cardiac fibrosis. Perindropil and modified citrus pectin (MCP) are correlated to the inhibition of myocardial fibrosis by acting on the myocardial Galectin-3 (Gal-3) gene (Dumic et al., 2006). In fact, a recent study, performed on rabbits affected by heart failure, has shown that a 4 weeks treatment with perindropil and MCP reduces the levels of Gal-3 and collagen type I and type III and reduces the fibrosis that follows myocardial infarction (Li et al., 2019). Stachydrine, is a pyrrolidine betaine, isolated from the seed and fruit of many Asian plants, that in a mouse model showed an antifibrotic activity by downregulating the AngII type 1 receptor and through the suppression of TGF-β1 in AngII stimulated cardiac fibroblasts (Liu X. et al., 2019). Chikusetsusaponin IVa (CS), is a triterpenoid saponin extracted from the roots of Panax Japonicus, that plays an important antifibrotic role by acting on autophagy. In fact, a study conducted on mice demonstrated that CS could improve the isoprenaline-induced myocardial fibrosis and reduced collagen deposition by the activation of autophagy via AMPK (Liu X. et al., 2019; Wang et al., 2019) (Figure 11).

## Conclusion

**FIGURE 12:**
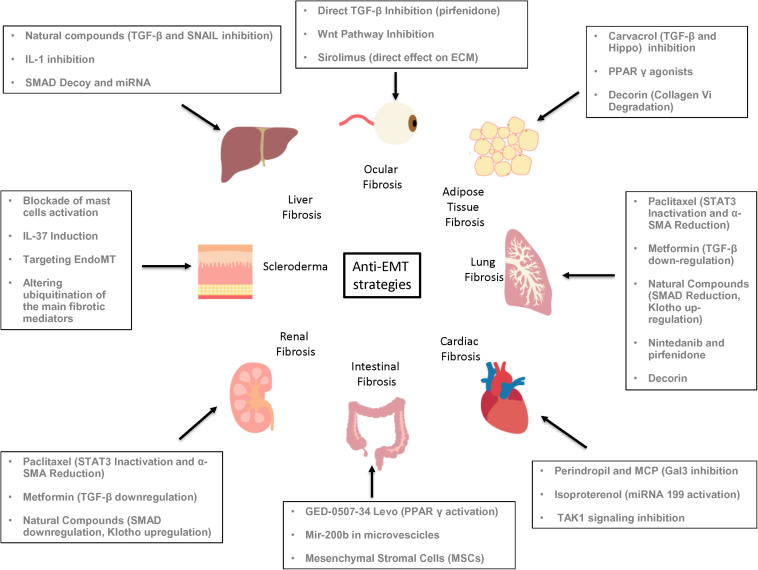
Summary of the possible anti-EMT therapeutic strategies. For each organ fibrosis are highlighted some of the most promising experimental drugs and therapeutic approaches that may block or revert the EMT process, thus controlling the fibrotic disease.

The uncontrolled fibrosis is a complex disease that can be mediated by a large number of mechanisms with molecular basis significantly different in different organs and tissues. This poses a serious obstacle to the development of a broadly effective anti-fibrotic therapy. In fact, currently, a unique treatment effective for all of the fibrotic disease is not yet available. However, in this review we have reported that a common feature shared, more or less strictly, among the fibrotic diseases is the EMT process. On the basis of this feature, we believe that targeting of the molecular mechanisms underlining EMT may provide new possibilities for the development of a broadly effective anti-fibrotic therapy (Figure 12).

## Author Contributions

JD, IR, and VF were responsible for assembling and drafting of the manuscript. All the other authors contributed to the drafting of the manuscript. VF edited and provided guidance for the assembling of the manuscript. All authors contributed to the article and approved the submitted version.

## Conflict of Interest

The authors declare that the research was conducted in the absence of any commercial or financial relationships that could be construed as a potential conflict of interest. The reviewer GL declared a shared affiliation with one of the authors, VF, to the handling editor at the time of review.
